# Wnt/Wingless signaling promotes lipid mobilization through signal-induced transcriptional repression

**DOI:** 10.1073/pnas.2322066121

**Published:** 2024-07-05

**Authors:** Mengmeng Liu, Rajitha-Udakara-Sampath Hemba-Waduge, Xiao Li, Xiahe Huang, Tzu-Hao Liu, Xianlin Han, Yingchun Wang, Jun-Yuan Ji

**Affiliations:** ^a^Department of Biochemistry and Molecular Biology, Louisiana Cancer Research Center, Tulane University School of Medicine, New Orleans, LA 70112; ^b^Lewis-Sigler Institute of Integrative Genomics, Princeton University, Princeton, NJ 08540; ^c^State Key Laboratory of Molecular Developmental Biology, Department of Molecular Systems Biology, Institute of Genetics and Developmental Biology, Chinese Academy of Sciences, Beijing 100101, China; ^d^Barshop Institute for Longevity and Aging Studies, University of Texas Health Science Center at San Antonio, San Antonio, TX 78229

**Keywords:** Wnt/Wg, lipolysis, transcriptional repression, lipid droplets, *Drosophila*

## Abstract

The Wnt/Wingless signaling pathway plays critical roles in metazoan development and energy metabolism, but its role in regulating lipid homeostasis remains not fully understood. Here, we report that the activation of canonical Wnt/Wg signaling promotes lipolysis while concurrently inhibiting lipogenesis and fatty acid β-oxidation in both larval and adult adipocytes, as well as cultured S2R+ cells, in *Drosophila*. Using RNA-sequencing and CUT&RUN (Cleavage Under Targets & Release Using Nuclease) assays, we identified a set of Wnt target genes responsible for intracellular lipid homeostasis. Notably, active Wnt signaling directly represses the transcription of these genes, resulting in decreased de novo lipogenesis and fatty acid β-oxidation, but increased lipolysis. These changes lead to elevated free fatty acids and reduced triglyceride (TG) accumulation in adipocytes with active Wnt signaling. Conversely, downregulation of Wnt signaling in the fat body promotes TG accumulation in both larval and adult adipocytes. The attenuation of Wnt signaling also increases the expression of specific lipid metabolism-related genes in larval adipocytes, wing discs, and adult intestines. Taken together, these findings suggest that Wnt signaling-induced transcriptional repression plays an important role in regulating lipid homeostasis by enhancing lipolysis while simultaneously suppressing lipogenesis and fatty acid β-oxidation.

## Significance

The Wnt signaling pathway, often deregulated in cancer and other diseases, remains poorly understood in lipid metabolism, particularly lipid mobilization. This study reveals that active Wnt/Wg signaling potently reduces lipid accumulation in *Drosophila* adipocytes by boosting lipolysis and inhibiting lipogenesis and fatty acid β-oxidation. Key Wnt target genes controlling fat storage and mobilization were identified; active Wnt signaling suppresses their transcription, while reduced Wnt signaling enhances it, thereby modulating triglyceride metabolism. Furthermore, active Wnt signaling directly represses the transcription of these lipid metabolism-related genes. These findings suggest that Wnt signaling-induced transcriptional repression regulates lipid homeostasis, balancing triglyceride storage and mobilization in *Drosophila* adipocytes.

The Wnt/Wingless (Wg) signaling pathway has long been recognized for its critical role in regulating metazoan development, with its deregulation linked to various cancers, particularly colorectal cancer (1–6). The core components of the canonical Wnt/Wg signaling pathway are evolutionarily conserved in *Drosophila*. In the absence of Wnt ligand binding, the β-catenin destruction complex, composed of the scaffold proteins Adenomatosis Polyposis Coli and Axin (Axn), together with the protein kinases Casein-kinase 1α and Glycogen Synthase Kinase 3, phosphorylates the transcriptional cofactor β-catenin (Armadillo/Arm in *Drosophila*), leading to its degradation in the cytoplasm. Consequently, *Drosophila* T-cell factor/Pangolin (dTCF/Pan), the T-cell factor (TCF) and lymphoid-enhancing factor (LEF) homolog in *Drosophila*, is repressed by transcription corepressors such as Groucho (Gro), a *Drosophila* Transducin-like Enhancer of split (TLE) homolog. This repression inactivates the canonical Wnt signaling pathway (6–8). Upon Wnt ligand binding, the destruction complex can no longer phosphorylate β-catenin/Arm, leading to the accumulation of β-catenin/Arm, which subsequently binds to dTCF/Pan, thereby activating the expression of Wnt target genes (7–9). The transition from Gro/TLE-mediated repression to Arm/β-catenin-driven activation involves Gro/TLE ubiquitylation by the HECT E3 ligase Hyd/UBR5, leading to the subsequent inactivation of the Gro/TLE corepressor (10). However, there are reports indicating that active Wnt signaling may directly repress the transcription of certain target genes, though the underlying mechanisms are still poorly understood (11).

In contrast to the extensively studied role of Wnt signaling in normal development and its recognized dysregulation in human cancers, its involvement in metabolic processes, particularly lipid metabolism, remains less understood (12). In mammals, Wnt signaling inhibits adipocyte differentiation, or adipogenesis (13, 14). Its role in regulating intricate processes such as lipolysis, β-oxidation of fatty acids (FAs), and lipogenesis remains a major unanswered question. The interconnected nature of these processes in mammals, along with multiple paralogs of key factors with partially redundant functions, complicates the study. While the fundamental metabolic processes are remarkably conserved in vertebrates, including glycolysis, the tricarboxylic acid (TCA) cycle, lipogenesis, lipolysis, and fatty acid β-oxidation (FAO), they are simpler in invertebrates such as *Drosophila* (15, 16). Notably, in *Drosophila*, adipogenesis only occurs during the late embryonic stage, temporally separated from processes such as lipogenesis, lipolysis, and FAO, which primarily occur during postembryonic stages (15, 17, 18). Hence, *Drosophila* serves as an ideal model system to elucidate the role of Wnt signaling in regulating these different aspects of lipid homeostasis.

We previously reported that homozygous *Axn^127^* mutant *Drosophila* larvae have defects in fat accumulation due to activated canonical Wnt signaling, which can be rescued by overexpressing α-catenin or treating with peptide boronic acids, such as Bortezomib (BTZ), Delanzomib, and Ixazomib (19). Moreover, α-catenin proves to be essential for the suppressive effects of these peptide boronic acids in mitigating Wnt signaling and rescuing the defective fat accumulation in *Axn^127^* mutant larvae (19). However, the mechanism by which Wnt signaling regulates lipid homeostasis remains poorly understood.

Building upon our previous work (19), this study shows that depleting negative regulators of Wnt signaling, such as *Axn* and *slmb* (*supernumerary limbs*), represses the expression of lipid metabolism-related genes, leading to fat accumulation defects similar to *Axn^127^* mutants. Conversely, down-regulating Wnt signaling increases the expression of these genes in adipocytes, wing discs, and adult intestines. Through molecular, proteomic, and genetic analyses, we identify a three-pronged mechanism by which active Wnt signaling reduces triglyceride (TG) accumulation and increases free fatty acid (FFA) levels: promoting lipolysis, reducing lipogenesis, and attenuating FAO in peroxisome and mitochondria. This involves direct repression of the transcription of lipid droplet-associated proteins (LDAPs) and key enzymes involved in lipogenesis and FAO. Collectively, our findings underscore the direct role of Wnt signaling in regulating lipid homeostasis, establishing a paradigm for Wnt signaling-induced transcriptional repression in regulating lipid homeostasis in *Drosophila*.

## Results

### Wnt/Wg Signaling Promotes Lipid Mobilization and Reduces Fat Accumulation.

**Fig. 1.**
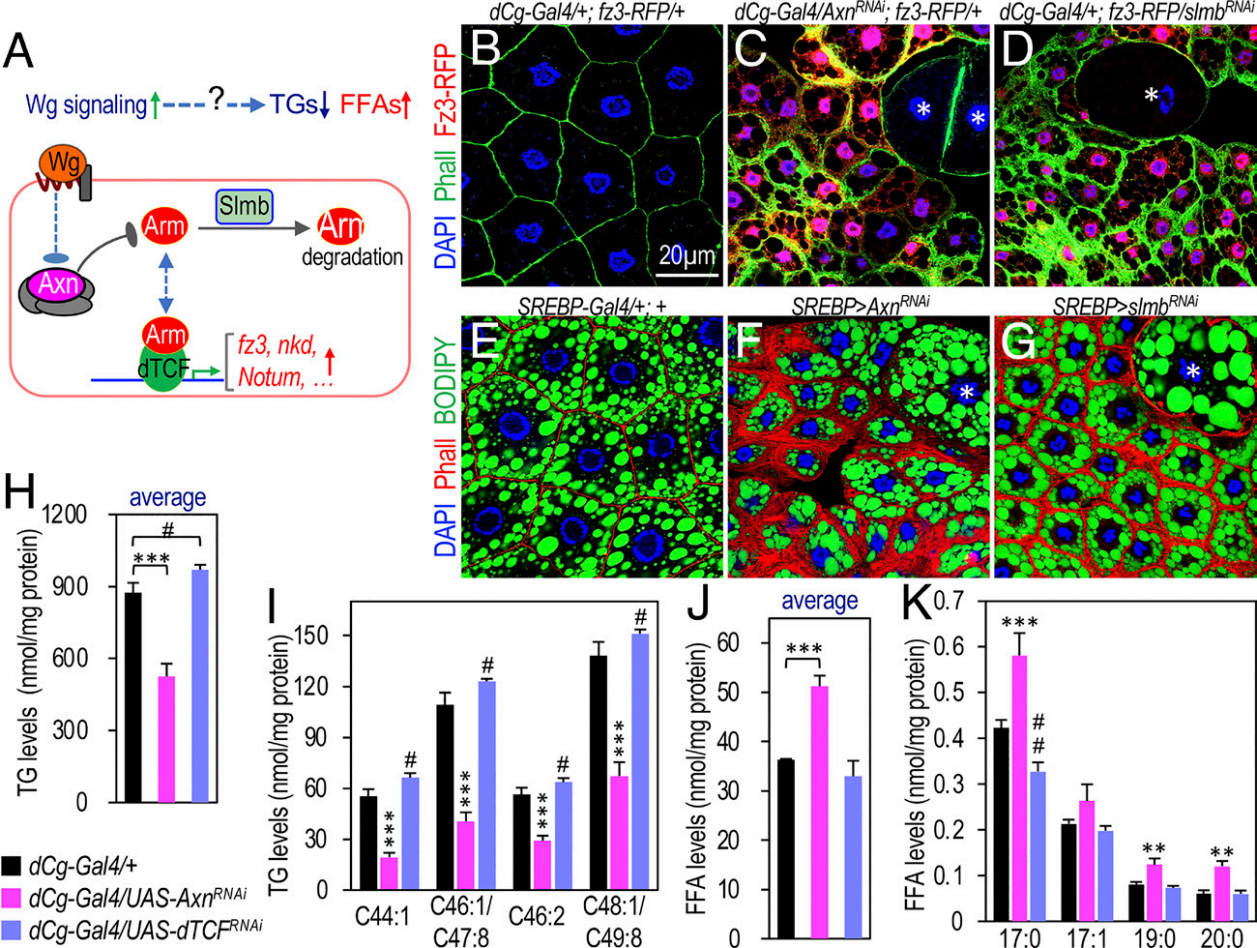
Wnt signaling regulates lipid homeostasis in larval adipocytes. (*A*) Schematic model illustrating how active Wnt signaling stimulates target gene expression. (*B*–*D*) Representative confocal images of larval adipocytes stained with DAPI (blue; nuclei) and Phall (Phalloidin, green; microfilament bundles). *fz3-RFP* expression (red) is increased in small adipocytes in (*C* and *D*) but low or absent expression in control (*B*) and large adipocytes (asterisks in *C* and *D*). Genotypes: (*B*) *dCg-Gal4/+; fz3-RFP/+*; (*C*) *dCg-Gal4/UAS-Axn^RNAi^; fz3-RFP*/+; and (*D*) *dCg-Gal4/+; fz3-RFP*/*UAS-slmb^RNAi^.* (*E*–*G*) Confocal images of larval adipocytes stained with DAPI (blue), Phall (red), and BODIPY (green; lipid droplets). Large adipocytes are marked with asterisks (*F* and *G*). Genotypes: (*E*) *SREBP-Gal4/+; +*; (*F*) *SREBP-Gal4/UAS-Axn^RNAi^; +*; and (*G*) *SREBP-Gal4/+; UAS-slmb^RNAi^/+.* (Scale bar in panel *B*: 20 μm.) (*H*–*K*) Quantitative lipidomics of TGs and FFAs in third instar wandering stage larvae. Average TG levels (*H*) and FFA levels (*J*) are shown. Representative TGs (*I*) and selected FFAs (*K*) are presented. Genotypes, color-coded for clarity, are listed below panel *H*. Asterisks (*) denote comparisons between “*dCg-Gal4/+*” and “*dCg-Gal4/UAS-Axn^RNAi^*”; pound signs (#) indicate comparisons between “*dCg-Gal4/+*” and “*dCg/UAS-dTCF^RNAi^*”. *P < 0.05; **P < 0.01; and ***P < 0.001, based on one-tailed unpaired *t* tests.

To investigate how active Wnt signaling reduces TG accumulation while concurrently increasing the levels of FFAs (Fig. 1*A*), we used a transgenic RNA interference (RNAi) approach to deplete either *Axn* or *slmb*, encoding a conserved E3 ubiquitin ligase required for the degradation of Arm (20). As expected, silencing *Axn* or *slmb* in larval adipocytes significantly increased the levels of Arm (*SI Appendix*, Fig. S1 *A*–*C*), which correlated to elevated expression of *fz3-RFP* (Fig. 1 *B*–*D*) and *nkd^EGFP^* (*SI Appendix*, Fig. S1 *D* and *D’* and *E* and *E’*). The expression of *fz3-RFP* and *nkd* is directly stimulated by Wnt signaling (21, 22). Interestingly, we observed heterogeneity in the size of adipocytes, with predominantly small adipocytes interspersed with occasional large ones (Fig. 1 *C*, *D*, *F*, and *G*). Smaller adipocytes exhibit higher levels of nuclear Arm (*SI Appendix*, Fig. S1 *B* and *C*) and increased expression of *fz3-RFP* (Fig. 1 *C* and *D*) and *nkd^EGFP^* (*SI Appendix*, Fig. S1 *E* and *E’*), suggesting that Wnt signaling was more active in small adipocytes compared to the adjacent larger adipocytes.

Importantly, the depletion of either *Axn* or *slmb* using multiple Gal4 lines active in adipocyte reduced adipocyte size, the number of lipid droplets, and TG levels (Fig. 1 *E*–*G* and *SI Appendix*, Fig. S1 *F*–*K*; quantified in *SI Appendix*, Fig. S1*L*). These Gal4 lines are located on different chromosomes, facilitating genetic recombination. Notably, these effects were rescued by depleting *dTCF* (*SI Appendix*, Fig. S1*N* cf. *SI Appendix*, Fig. S1*K*, control shown in *SI Appendix*, Fig. S1*M*). These observations suggest that the canonical Wnt signaling is particularly active in small adipocytes, resulting in reduced fat accumulation, consistent with its role in disrupting fat accumulation in larval adipocytes (19). Furthermore, the depletion of *Axn* in adult adipocytes also significantly reduced TG accumulation (*SI Appendix*, Fig. S2 *A*–*C*), suggesting that the effect of active Wnt signaling on lipid metabolism is not limited to the larval stage.

To analyze the physiological role of Wnt signaling on lipid metabolism, we performed lipidomics analyses on whole larvae after depleting *dTCF* in the larval fat body and inducing Wnt signaling by depleting *Axn* in the fat body as a control. Consistent with our prior report demonstrating that hyperactive canonical Wnt signaling in *Axn^127^* mutant larvae led to a general reduction in TG levels and an increase in FFAs (19), the targeted activation of Wnt signaling in the fat body significantly decreased TG levels (Fig. 1*H*) while elevating FFAs (Fig. 1*J*). Specifically, Wnt signaling activation led to a notable reduction in 33 out of 71 analyzed TG types (Fig. 1*I* and *SI Appendix*, Fig. S3 *A*–*D*). Concurrently, nine out of the 15 detected FFAs showed a significant increase (Fig. 1*K* and *SI Appendix*, Fig. S3 *E* and *F*). Although the impact on FFAs was milder (Fig. 1 *J* and *K*) compared to the *Axn^127^* mutant larvae (19), likely due to the weaker effects of *Axn* depletion via the transgenic RNAi approach, these results extend our previous findings of hyperactive Wnt signaling contributing to reduced TG accumulation. Importantly, the downregulation of Wnt signaling yielded opposite effects, increasing TG levels (Fig. 1 *H* and *I* and *SI Appendix*, Fig. S3 *A*–*D*) while simultaneously diminishing specific FFAs (Fig. 1*K* and *SI Appendix*, Fig. S3 *E* and *F*). Among the 71 analyzed TG types, 29 exhibited a significant increase following Wnt signaling downregulation (Fig. 1*I* and *SI Appendix*, Fig. S3 *A*–*D*), whereas three out of 15 FFAs showed a significant decrease (Fig. 1*K* and *SI Appendix*, Fig. S3 *E* and *F*).

Notably, the lipidomics analyses were conducted on mid-wandering stage larvae, approximately 88 h after hatching. Within this limited timeframe, various processes must unfold, including the time required for the Gal4-UAS system and RNAi to deplete dTCF mRNA, effects on Wnt target gene expression, and the dynamic changes in lipid metabolism. Thus, the data from this experiment may potentially underestimate the actual impact. To further assess the physiological relevance of Wnt signaling in regulating lipid metabolism, we depleted either Arm or dTCF in female flies for five or 30 d. We observed a significant increase in TG levels (*SI Appendix*, Fig. S2 *D* and *E*). These observations collectively underscore the physiological importance of Wnt signaling in regulating lipid homeostasis.

### Active Wnt Signaling Regulates Lipid Homeostasis in Cultured S2R+ Cells.

To test whether the reduced lipid accumulation observed in adipocytes resulted from increased lipolysis or reduced lipogenesis, we used dual radioisotope labeling with ^14^C-labeled glucose and ^3^H-palmitic acid. This approach allowed us to evaluate the effects of Wnt signaling on the breakdown and synthesis of FFAs and TGs. In this dual labeling scheme, ^14^C-glucose is metabolized through glycolysis to yield acetyl-CoA, which can be either oxidized to CO_2_ via the TCA cycle or utilized for biosynthesis of FFAs and TGs through lipogenesis. On the other hand, ^3^H-palmitic acid can undergo direct esterification with glycerol, potentially incorporating ^14^C-labeling, to produce TGs or be subjected to oxidization via β-oxidation, producing acetyl-CoA that can be further oxidized to CO_2_. Thus, the relative rates of ^14^C and ^3^H accumulation, as expressed by the ^14^C/^3^H ratio, in TGs and FFAs offer a measure of the rate of lipolysis or lipogenesis. A decreased ^14^C/^3^H ratio indicates primarily increased lipolysis, whereas an increased ratio indicates the opposite (23–26).

**Fig. 2.**
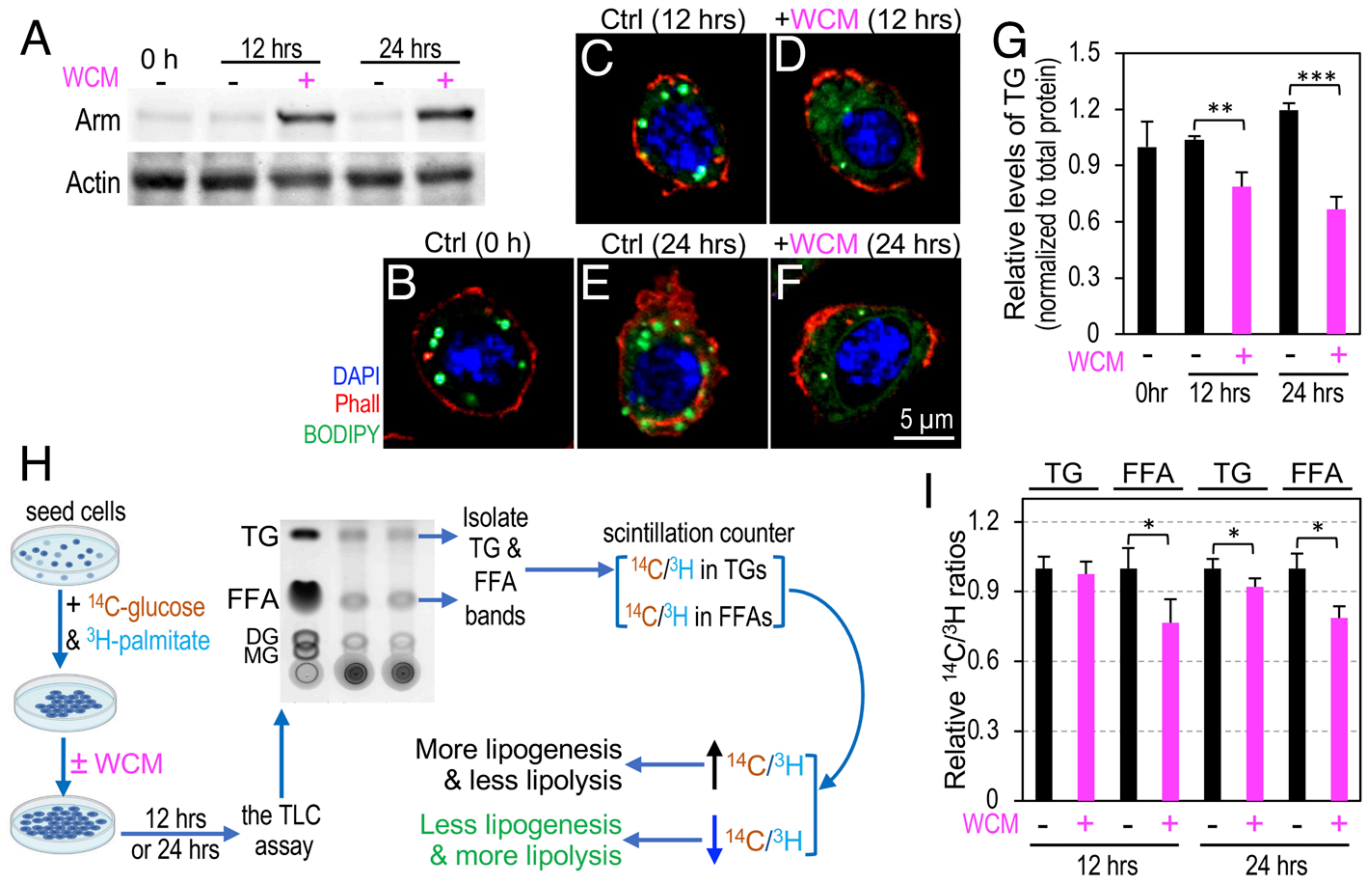
Active Wnt signaling regulates lipid homeostasis in cultured S2R+ cells. (*A*) Western blot analysis of Arm protein in S2R+ cells treated with WCM (Wg-conditioned medium). Actin serves as a loading control. (*B*–*F*) Confocal images of S2R+ cells stained with DAPI (blue), Phall (red), and BODIPY (green). Cells were treated with WCM for 12 h (*D*) or 24 h (*F*). (Scale bar in *F*: 5 μm.) (*G*) Quantification of TG levels, normalized to the control (n = 3, independent biological repeats). (*H*) Experimental design for dual-isotope radiolabeling experiments using S2R+ cells. TLC: thin layer chromatography; DG: diacylglyceride; MG: monoacylglyceride. Lipids from S2R+ cell pellets were loaded onto TLC lanes and normalized to total protein levels. (*I*) Relative ^14^C/^3^H ratios in TG and FFAs of S2R+ cells treated with WCM for 12 h or 24 h.

To minimize compounding factors, we used cultured *Drosophila* S2R+ cells, known for their lipid droplet accumulation (27–29), instead of larvae fed with fly food containing vaguely defined, carbohydrate-rich ingredients. Depletion of *Axn* in S2 cells activates Wnt signaling (30), as does treating cultured *Drosophila* cell lines such as Kc167 cells, S2R+ cells (Fig. 2*A*), or cl-8 cells with Wg-conditioned medium (WCM) (8, 31, 32). WCM contains Wg protein secreted from cultured S2-Tub-Wg cells. Notably, treatment with WCM not only activates Wnt signaling but also reduces the number of lipid droplets and the level of TGs (Fig. 2 *B*–*F*; quantified in Fig. 2*G*). These results suggest that the effect of active Wnt signaling in reducing TG accumulation is not limited to adipocytes. We supplemented the medium with ^14^C-labeled glucose and ^3^H-palmitic acid, followed by treating S2R+ cells with WCM to activate Wnt signaling. Subsequently, we used thin-layer chromatography (TLC) to separate TGs and FFAs, and their ^14^C/^3^H ratios were quantified using liquid scintillation (Fig. 2*H*). This analytical approach revealed a significant reduction in the ^14^C/^3^H ratios of both FFAs and TGs in S2R+ cells after the WCM treatment (Fig. 2*I*). These findings constitute direct evidence supporting the role of Wnt signaling in promoting lipolysis, inhibiting de novo lipogenesis, or potentially both processes simultaneously, irrespective of adipogenesis.

### Wnt Signaling Represses the Transcription of Genes Encoding Factors that Control Intracellular Lipid Homeostasis.

**Fig. 3.**
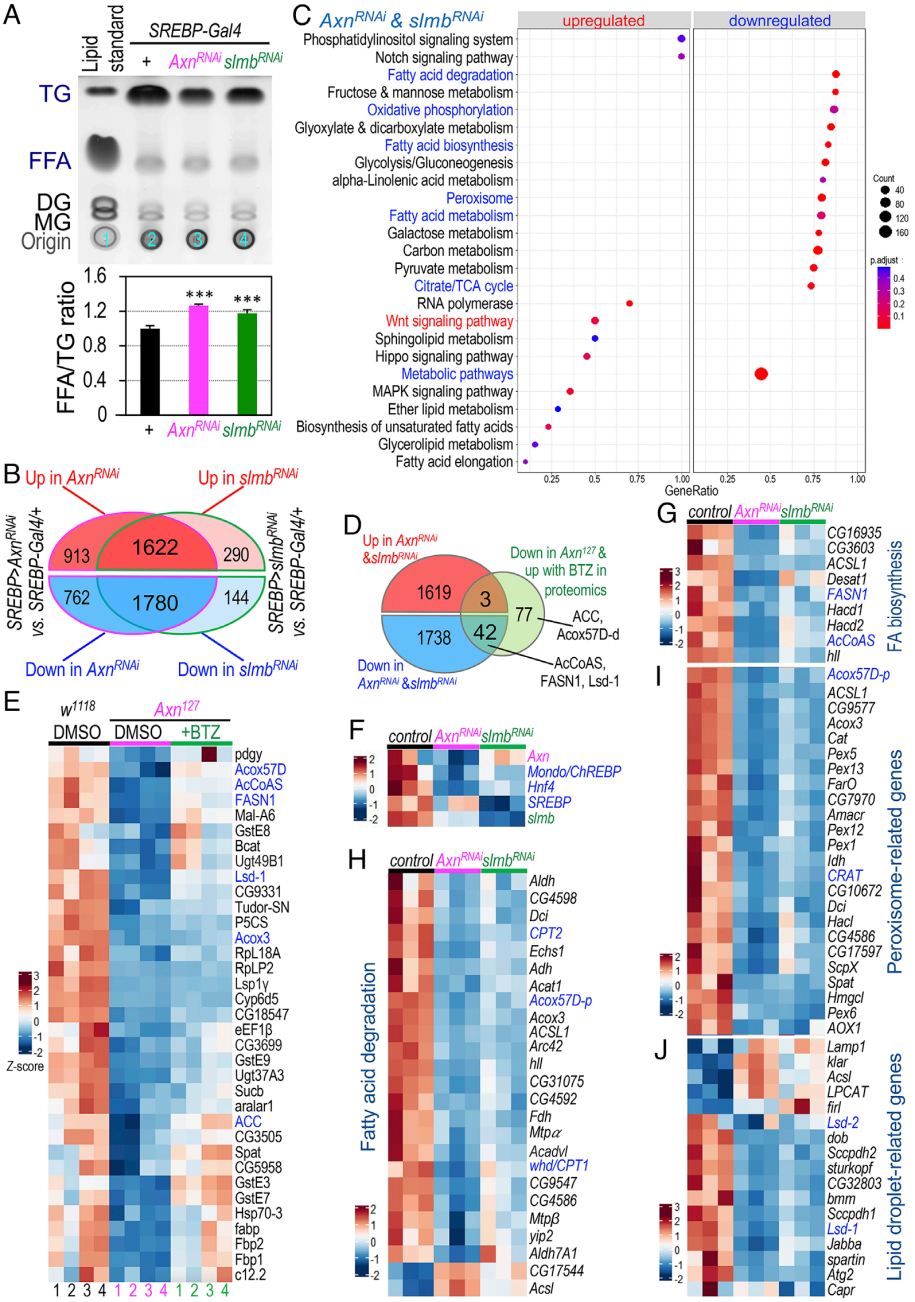
Wnt signaling represses the transcription of lipid metabolism related genes. (*A*) Relative levels of FFAs and TGs assessed by TLC assay in larvae with fat body-specific depletion of either *Axn* or *slmb*. Genotypes: *SREBP-Gal4/+; +* (lane #2); *SREBP-Gal4/UAS-Axn^RNAi^; +* (lane #3); and *SREBP-Gal4/+; UAS-slmb^RNAi^/+* (lane #4). Lower panel quantifies FFA/TG ratios from three independent biological repeats (***P < 0.01, one-tailed unpaired *t* tests). (*B*) Venn diagram showing genes altered in fat body with *Axn* or *slmb* depletion. (*C*) Commonly affected metabolic pathways in larval fat body with *Axn* or *slmb* depletion. Down-regulated pathways linked to carbohydrate and lipid metabolism (blue); Wnt signaling pathway (red, significantly up-regulated). (*D*) Venn diagram of genes altered in *Axn^RNAi^* or *slmb^RNAi^* fat body, compared to proteins down-regulated in *Axn^127^* mutant larvae but reversed by feeding larvae with 2 μM BTZ. (*E*) Heatmap of proteins reduced in *Axn^127^* mutant larvae but reversed by BTZ, quantified by proteomics. Key proteins are highlighted in blue. (*F*) Heatmap of mRNA levels of *Mondo/ChREBP*, *Hnf4*, and *SREBP* in *Axn^RNAi^* or *slmb^RNAi^* fat body; mRNA levels of *Axn* and *slmb* serve as positive controls. (*G*–*J*) Heatmaps of gene expression levels in metabolic pathways: “fatty acid biosynthesis” (*G*), “fatty acid degradation” (*H*), “peroxisome” (*I*), and “lipid droplet-related” genes (*J*). Data shown as z-scores based on normalized counts across triplicates per genotype. Key genes further analyzed are highlighted in blue.

Our previous analyses of the *Axn^127^* homozygous mutant larvae revealed that hyperactive Wnt signaling caused a general reduction of most types of TGs while simultaneously increasing the levels of different FFAs (19). These effects were further validated in larvae with adipocyte-specific activation of Wnt signaling (Fig. 3*A*). To gain insights into the molecular mechanism underlying Wnt signaling-stimulated lipid catabolism and reduced lipogenesis, we conducted bulk RNA sequencing (RNA-seq) analyses on dissected larval fat body with specific depletions of *Axn* or *slmb*. Our analysis identified a total of 1,622 up-regulated genes and 1,780 down-regulated genes common to both *Axn^RNAi^* and *slmb^RNAi^* adipocytes when compared to the controls (Fig. 3*B*; both adjusted p- and q-values were less than 0.05). As expected, our “Pathway” and “Gene Ontology” cluster analyses (33) identified the significant activation of the “Wnt signaling pathway” in both *Axn^RNAi^* and *slmb^RNAi^* fat bodies (Fig. 3*C*). Among the up-regulated genes in this pathway were direct Wnt target genes, including *fz3* (*frizzled 3*), *nkd* (*naked cuticle*), *Notum*, and others (*SI Appendix*, Fig. S4*A*). Active Wnt signaling also significantly affected multiple biological pathways, leading to either upregulation or downregulation (Fig. 3*C* and *SI Appendix*, Fig. S4 *B* and *C*). Remarkably, several lipid metabolism-related pathways were significantly down-regulated in adipocytes with active Wnt signaling, including “FA degradation,” “FA biosynthesis,” “FA metabolism,” “oxidative phosphorylation,” “TCA cycle” and “peroxisome” pathways (Fig. 3*C*).

Given that the absolute steady-state levels of mRNAs may not always precisely correlate with the cellular abundance of their corresponding proteins (34), we performed quantitative proteomic analyses using TMT (tandem mass tag) labeling (35–37). Previously, we reported that feeding *Axn^127^* mutant larvae with peptide boronic acids, such as BTZ, could stabilize α-catenin (19). This stabilization facilitated the sequestration of excess β-catenin beneath the cell membrane, thereby down-regulating canonical Wnt signaling in *Axn^127^* mutants and rescuing defects in fat accumulation (19). Therefore, we analyzed the protein levels in *Axn^127^* mutant larvae and the effects of BTZ treatment. Next, we compared the list of proteins to the list of genes with significantly altered mRNA levels in *Axn^RNAi^* and *slmb^RNAi^* fat bodies. This comparison led us to identify 45 proteins whose levels changed in concordance with their mRNA levels (Fig. 3*D*). Among these 45 proteins, 42 exhibited significant decreases in *Axn^127^* mutants yet displayed upregulation upon BTZ treatment (Fig. 3*D*). Five notable proteins among the 42 are FASN1 (FA synthase 1) and ACC (acetyl-CoA carboxylase), crucial for TG biosynthesis; AcCoAS (acetyl Coenzyme A synthase), bridging FA synthesis and the TCA cycle; Acox57D (acyl-CoA oxidase at 57D), involved in peroxisomal FAO; and Lsd-1 (Lipid storage droplet-1), the *Drosophila* homolog of Perilipin-1, associated with lipid droplets (LDs) (Fig. 3*E*). These results indicate that active Wnt signaling potentially regulates intracellular lipid homeostasis through certain key factors involved in FA synthesis, FAO, and LDAPs present on LDs.

Due to the limitations of quantitative proteomic analyses in detecting proteins with low abundance in larvae, we further analyzed the RNA-seq data by focusing on genes involved in FA biosynthesis, FAO, LDAPs, and peroxisome-related genes. This detailed analysis revealed a consistent reduction in the expression of genes encoding crucial enzymes and factors associated with FA biosynthesis and degradation when Wnt signaling was active (Fig. 3 *F*–*H* and *SI Appendix*, Fig. S4*D*). Remarkably, the significant reduction in mRNA levels of key transcriptional factors in *Axn^RNAi^* and *slmb^RNAi^* fat bodies, such as *SREBP*, *Mondo* (the *Drosophila* homolog of carbohydrate-responsive element-binding protein, ChREBP), and *dHnf4* (Fig. 3*F*), is of particular interest. This reduction of *SREBP* and *Mondo/ChREBP* correlates with the decreased expression of their target genes, including *FASN1*, *AcCoAS*, and *ACSL1* (long-chain fatty acid-CoA ligase) (Fig. 3*G*). Likewise, the reduced *dHnf4* mRNA levels are associated with a notable decrease in *whd* (*withered*, which encodes carnitine O-palmitoyltransferase CPT1), *CPT2*, *CG4598*, and *yip2* (Fig. 3*H*). The transcription of these genes is regulated by dHnf4 in *Drosophila* (38). CPT1 and CPT2 are required for transporting fatty acids into the mitochondria for FAO (39–41), while *CG4598* and *yip2* encode enzymes involved in mitochondrial FAO (38). Collectively, these findings suggest that active Wnt signaling significantly attenuates mitochondrial FA degradation in larval adipocytes. In addition to the mitochondria, peroxisomes also play a critical role in β-oxidation of FAs (40–42). Active Wnt signaling also reduced the expression of *CRAT* (Fig. 3*I*), which encodes carnitine O-acetyl-transferases responsible for transporting very long-chain FAs into peroxisomes. Furthermore, multiple genes encoding Peroxins and other peroxisomal proteins exhibited reduced expression levels (Fig. 3*I* and *SI Appendix*, Fig. S4*E*). The downregulation of these key factors central to FAO in *Axn^RNAi^* and *slmb^RNAi^* adipocytes suggests that Wnt signaling negatively regulates FAO in both peroxisomes and mitochondria. Consistent with the dual radioisotope labeling assay results conducted in S2R+ cells (Fig. 2*I*), these observations in adipocytes support the notion that active Wnt signaling inhibits both de novo lipogenesis and FAO processes.

The identification of Lsd-1 in our proteomic analyses prompted us to broaden our investigation to include other LDAPs. In addition to *Lsd-1*, we observed significant reductions in the expression of several genes encoding LDAPs, including *Lsd-2*, *Jabba*, *spartin*, and *sturkopf*, in *Axn^RNAi^* and *slmb^RNAi^* adipocytes (Fig. 3*J*). *Drosophila* has two perilipin orthologs, Lsd-1 (Perilipin-1) and Lsd-2 (Perilipin-2), which hinder lipolysis and lipid mobilization by preventing lipases from accessing stored TGs in LDs (43). Thus, the reduction in LDAP expression in adipocytes, induced by active Wnt signaling, may explain how Wnt signaling promotes lipolysis.

### Validating the Impact of Wnt Signaling on the Transcription of Specific Lipid Metabolism-Related Genes.

**Fig. 4.**
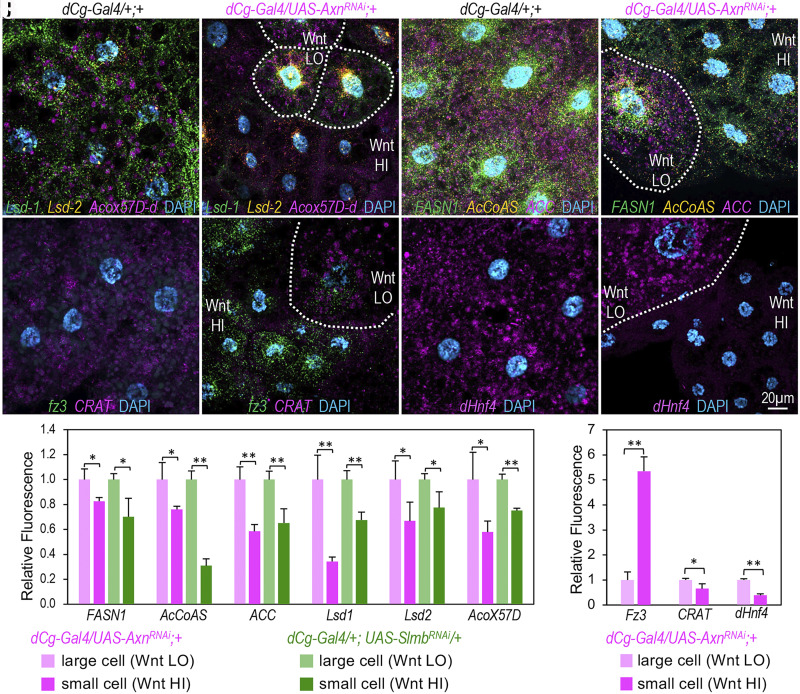
Wnt signaling-regulated gene transcription in regulating lipid homeostasis. (*A* and *B*) HCR RNA-FISH assay detects mRNA transcripts of *Lsd-1* (green), *Lsd-2* (orange), and *Acox57D-d* (magenta) in larval adipocytes. Large adipocytes with low Wnt/Wg activity are labeled “Wnt LO” (low Wnt signaling). Adjacent small adipocytes with active Wnt/Wg signaling are labeled “Wnt HI” (high Wnt signaling). (*C* and *D*) HCR RNA-FISH assay visualizes mRNA transcripts of *FASN1* (green), *AcCoAS* (orange), and *ACC* (magenta) in adipocytes, showing lower transcript levels in small adipocytes compared to large ones (*D*). (*E* and *F*) mRNA transcripts of *fz3* (green) and *CRAT* (magenta) detected by HCR assay in adipocytes. (*G* and *H*) HCR assay detects mRNA transcripts of *Hnf4* (magenta) in larval adipocytes. Probe sets: B1-Alexa Fluor 488 (green), B2-Alexa Fluor 594 (orange), and B3-Alexa Fluor 647 (magenta) amplifiers. Images are projections of 12 to 15 successive optical sections, spaced 1.0 μm apart. Genotypes: (*A*, *C*, *E*, and *G*) *dCg-Gal4/+; +*; and (*B*, *D*, *F*, and *H*) *dCg-Gal4/UAS-Axn^RNAi^; +*. (Scale bar in panel *H*: 20 μm.) (*I* and *J*) Quantification of mRNA transcripts (*P < 0.05; **P < 0.01, one-tailed unpaired *t* tests).

To validate the suppressive effect of Wnt signaling on the transcription of various genes—including *Lsd1*, *Lsd2*, *Acox57D-d*, *FASN1*, *AcCoAS*, *ACC*, *CRAT*, and *dHnf4*—at the cellular level, we analyzed their mRNA transcripts using multiplexed in situ hybridization chain reaction (HCR) RNA fluorescence in situ hybridization (RNA-FISH) imaging technology (44, 45). Our results from these analyses confirmed that the mRNA levels of *Lsd1*, *Lsd2*, and *Acox57D-d* (Fig. 4 *A* and *B* and *SI Appendix*, Fig. S5 *A*–*C*), as well as *FASN1*, *AcCoAS*, and *ACC* (Fig. 4 *C* and *D* and *SI Appendix*, Fig. S5 *D*–*F*), were significantly reduced in small adipocytes compared to the controls or the adjacent large adipocytes in *Axn^RNAi^* and *slmb^RNAi^* fat bodies (quantified in Fig. 4*I*). Similar observations were made with *CRAT* and *dHnf4*, using *fz3* as a positive control (Fig. 4 *E*–*H*; quantified in Fig. 4*J*). These observations, coupled with the results from RNA-seq and quantitative proteomic analyses (Fig. 3 *E*–*J*), collectively confirm that active Wnt signaling in adipocytes represses the transcription of genes related to lipogenic (*FASN1*, *AcCoAS*, and *ACC*), LDAPs (*Lsd1* and *Lsd2*), and factors involved in FAO (*dHnf4*, *CRAT*, and *Acox57D-d*).

While it remains uncertain whether these inhibitory effects are conserved in other species, it is noteworthy that the inhibitory effects of Wnt signaling on *FAS* gene expression and lipogenesis have been documented in mouse adipocytes and the liver of juvenile turbot treated with the GSK3 inhibitor Lithium chloride (46, 47). Given that the role of Wnt signaling in regulating lipolysis remains largely unexplored, our subsequent analyses were focused on the involvement of Wnt/Wg signaling in lipolysis and lipid mobilization.

### The Physiological Role of Wnt Signaling in Modulating the Expression of Lipid Metabolism-Related Genes in Different Cell Types and Developmental Stages.

Because the downregulation of Wnt signaling in adipocytes through the depletion of dTCF or Arm increased TG levels (Fig. 1 *H* and *I* and *SI Appendix*, Figs. S2 *D* and *E* and S3 *A*–*D*), we investigated the effects of reducing Wnt signaling on the expression of these lipid metabolism-related genes.

**Fig. 5.**
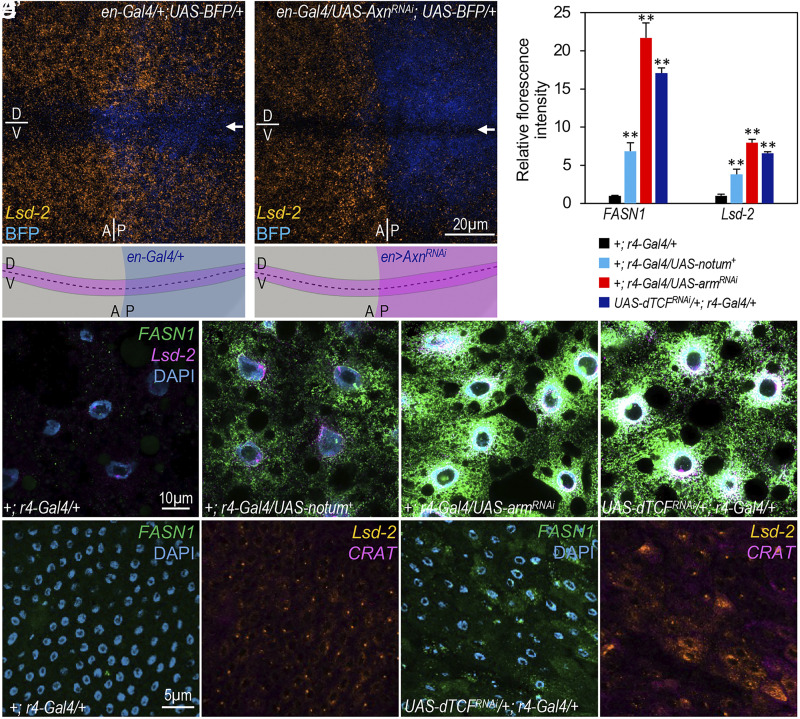
Physiological role of Wnt signaling in regulating lipid metabolism-related gene expression in different cell types. (*A* and *B*) Confocal images showing mRNA transcripts of *Lsd-2* (orange) in wing discs using the HCR assay, with BFP (blue fluorescent protein) shown in blue. Arrows indicate the dorsal/ventral (D/V) boundary with active and endogenous Wnt signaling. Diagrams below depict both the D/V and the anterior/posterior (A/P) boundaries of wing discs. (Scale bar in panel *B*: 20 μm.) Genotypes: (*A*) *en-Gal4/+; UAS-BFP/+*; and (*B*) *en-Gal4/UAS-Axn^RNAi^; UAS-BFP/+*. (*C*–*F*) Representative confocal images illustrating mRNA transcripts of *FASN1* (green) and *Lsd-2* (magenta) in larval adipocytes of indicated genotypes using the HCR assay. These images are single focal planes due to strong upregulation of *FASN1* and *Lsd-2*. Genotypes: (*C*) *+; r4-Gal4/+*; (*D*) *+; r4-Gal4/UAS-notum^+^*; (*E*) *+; r4-Gal4/UAS-arm^RNAi^*; and (*F*) *UAS-dTCF^RNAi^/+; r4-Gal4/+*. (*G*) Quantification of *FASN* and *Lsd-2* mRNA transcripts for the indicated genotypes (*C*–*F*). Scale bar in panel *C* corresponds to images (*C*–*F*): 10 μm. (*H* and *I*) Confocal images showing mRNA transcripts of *FASN1* (green), *Lsd-2* (orange), and *CRAT* (magenta) in the adult intestine of specific genotypes using the HCR assay. Genotypes: (*H* and *H’*) *+; r4-Gal4/+*; and (*I* and *I’*) *UAS-dTCF^RNAi^/+; r4-Gal4/+*. Scale bar in panel *H* corresponds to images (*H* and *H’* and *I* and *I’*): 5 μm.

Considering the known activation of endogenous Wnt signaling in the dorsal–ventral boundary of wing discs (48) (depicted in the diagram in Fig. 5*A*), we tested whether Wnt signaling represses the expression of target genes, such as *Lsd-1*, *Lsd-2*, and *Acox57D*, in wing discs. Consistent with a previous study (49), we observed a notable reduction in *Lsd-2* transcripts in cells along the dorsal–ventral boundary of wing discs (Fig. 5*A*), indicating normal repression of *Lsd-2* transcription by endogenous Wnt signaling. To further verify the causal relationship between Wnt signaling and reduced *Lsd-2* expression in wing disc cells, we depleted *Axn* in the posterior compartment of wing discs using *en-Gal4*, resulting in a significant reduction in the levels of *Lsd-2* transcripts in the posterior compartment (Fig. 5*B*; the images of separate channels are presented in *SI Appendix*, Fig. S6). The transcription of *Lsd-1* and *Acox57D* could not be detected in wing discs (*SI Appendix*, Fig. S6 *A”* and *A”’*), precluding an analysis of the effects of Wnt signaling on repressing their transcription in this context. These observations suggest that the inhibitory effect of active Wnt signaling on the transcription of certain target genes, such as *Lsd-2*, is not confined to larval adipocytes.

Encouraged by this observation, we genetically down-regulated Wnt signaling in larval adipocytes and analyzed the effects on the expression of key lipid metabolism related genes. Overexpression of Notum, which inactivates Wnt signaling by deacylating extracellular Wnt/Wg ligands (50, 51), using the *r4-Gal4* line significantly increased mRNA levels of *FASN1* and *Lsd-2* (Fig. 5*D*) compared to the control (Fig. 5*C*; quantified in Fig. 5*G*). Another strategy involved the ablation of either *β-catenin/arm* or *dTCF.* A more pronounced upregulation of *FASN1* and *Lsd-2* was observed upon depletion of either *arm* (Fig. 5*E*) or *dTCF* (Fig. 5*F*; quantified in Fig. 5*G*; the images of separate channels are presented in *SI Appendix*, Fig. S7), consistent with the repressive role of Wnt signaling in the transcriptional regulation of *FASN1* and *Lsd-2* (Figs. 3 and 4).

Given the activity of the *r4-Gal4* line in adult gut tissues (52), we explored whether down-regulating Wnt signaling in the adult intestine could alter the expression of lipid metabolism-related genes. Compared to the control (Fig. 5 *H* and *H’* and *SI Appendix*, Fig. S8*A*), a significant increase in the expression of *FASN1*, *Lsd-2*, and *CRAT* was observed in adult midgut with *dTCF* depletion (Fig. 5 *I* and *I’* and *SI Appendix*, Fig. S8*B*). Overexpressing *Notum* using *r4-Gal4* resulted in pupal lethality; thus, we also tested the effects of Wnt signaling inhibition caused by the overexpression of a dominant-negative form of *dTCF* (*UAS-dTcf^DN^*). A substantial upregulation of *FASN1*, *Lsd-2*, and *CRAT* was also observed in the intestines of these adults (*SI Appendix*, Fig. S8 *C*–*C’”*), compared to the control (Fig. 5 *H* and *H’* and *SI Appendix*, Fig. S8*A*).

In summary, these observations suggest that inhibiting or reducing Wnt signaling up-regulates genes associated with lipid metabolism, consistent with the observed increase in TG levels (Fig. 1 *H* and *I* and *SI Appendix*, Fig. S2). These data collectively support the inhibitory role of Wnt signaling in regulating the expression of these genes.

### Wnt Signaling Stimulates Lipolysis through LDAPs.

**Fig. 6.**
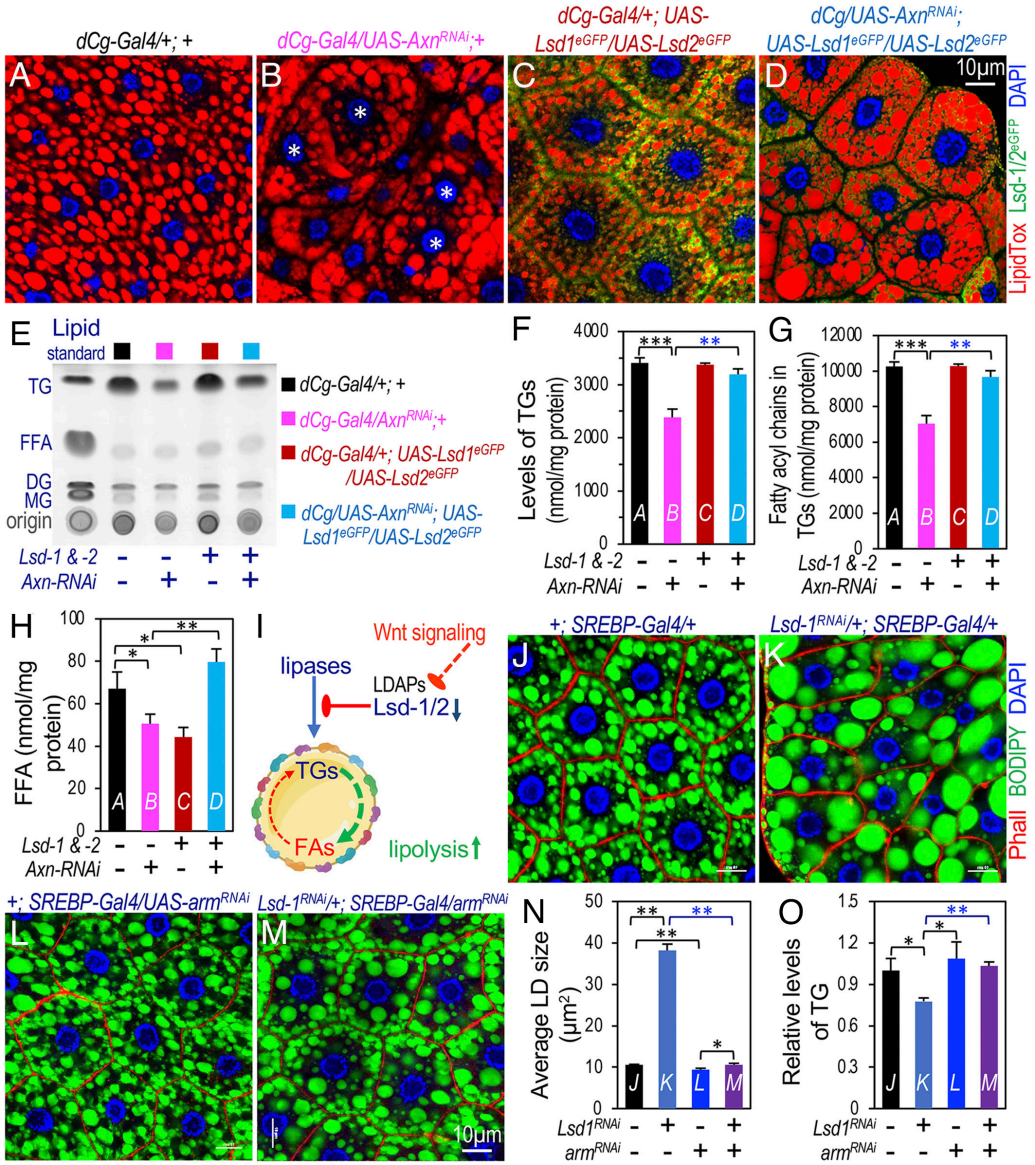
Interplay between LDAPs and Wnt signaling. (*A*–*D*) Representative confocal images of larval adipocytes stained with DAPI (blue), LipidTox (red), and GFP (green). Genotypes: (*A*) *dCg-Gal4/+; +*; (*B*) *dCg-Gal4/UAS-Axn^RNAi^;* (*C*) *dCg-Gal4/+; UAS-Lsd1^eGFP^/UAS-Lsd2^eGFP^*; and (*D*) *dCg-Gal4/UAS-Axn^RNAi^; UAS-Lsd1^eGFP^/UAS-Lsd2^eGFP^.* Additional controls and single-channel images are in *SI Appendix*, Fig. S9. (*E*) Chromatographic separation of lipids from indicated genotypes using TLC analysis. Lane 1: lipid standard mixture (MG, DG, TG, and stearic acid representing FFAs). (*F*–*H*) Quantitative lipidomics measurement of TGs (*F*), Fatty acyl chains in TGs (*G*), and FFAs (*H*) in fat bodies from third instar larvae of indicated genotypes, color-coded for clarity. (*I*) Schematic model illustrating how Wnt signaling stimulates lipolysis and lipid mobilization by inhibiting LDAP expression (e.g., *Lsd-1* and *Lsd-2*). (*J*–*M*) Confocal images of larval adipocytes, stained with DAPI (blue), Phall (red), and BODIPY (green). Genotypes: (*J*) *+; SREBP-Gal4/+*; (*K*) *UAS-Lsd1^RNAi^/+; SREBP-Gal4/+*; (*L*) *+; SREBP-Gal4/UAS-arm^RNAi^*; and (*M*) *UAS-Lsd1^RNAi^/+; SREBP-Gal4/UAS-arm^RNAi^*. (*N* and *O*) Quantification of average lipid droplet (LD) size (*N*) and TG levels (*O*) for indicated genotypes (n = 3 biological repeats). For TG quantification, samples were normalized based on total protein levels determined using the Bradford protein assay. Results were normalized to control (*+; SREBP-Gal4/+*). (Scale bar in panels *D* and *M*: 10 μm.)

The finding of Wnt signaling-induced repression of *Lsd-1* and *Lsd-2* prompted us to hypothesize that active Wnt signaling reduces the presence of LDAPs, including Lsd-1 and Lsd-2, on the surface of lipid droplets in adipocytes; this reduction may increase the access of lipases to TGs stored within lipid droplets, thereby stimulating lipid mobilization, converting TG, to diacylglycerides, monoacylglycerides, and eventually to FFAs (Fig. 6*I*). This model predicts that the overexpression of LDAPs should mitigate the effects of Wnt signaling on lipid mobilization.

Given that Lsd-1 and Lsd-2 exhibit partially redundant roles in lipid metabolism (53, 54), we tested whether overexpressing Lsd-1, Lsd-2, or both could rescue adipocyte defects resulting from active Wnt signaling. Compared to the larval fat body depleted of *Axn* (Fig. 6*B*; with the heterozygous control for *dCg-Gal4* shown in Fig. 6*A*), the overexpression of either *Lsd-1* or *Lsd-2* alone partially mitigated the adipocyte defects caused by active Wnt signaling (*SI Appendix*, Fig. S9 *B* and *D*, with controls shown in *SI Appendix*, Fig. S9 *A* and *C*). Remarkably, the simultaneous overexpression of both *Lsd-1* and *Lsd-2* strongly rescued the fat-body defects induced by Wnt signaling (Fig. 6*D* cf. Fig. 6*B*; single-channel images of these data are shown in *SI Appendix*, Fig. S9). Using TLC analysis to quantify these effects, we found that the overexpression of both *Lsd-1* and *Lsd-2* strongly rescued the effects of *Axn* depletion on TG levels (Fig. 6*E*).

To quantify these effects on different types of lipids precisely, we dissected larval fat bodies from these genotypes and conducted lipidomic analyses. As expected, the decreased TG levels in *Axn*-depleted adipocytes were effectively rescued by the overexpression of *Lsd-1* and *Lsd-2* (Fig. 6*F*). In addition, the fatty acyl chains in TGs, which represents the composition of each fatty acyl chain within the total TG pool, were reduced by active Wnt signaling, while the overexpression of *Lsd-1* and *Lsd-2* significantly rescued these defects (Fig. 6*G*). However, the level of FFAs in *Axn^RNAi^* adipocytes appeared to be reduced in this analysis (Fig. 6*H*). These results differed from analyses conducted using whole larvae (19); a likely explanation is that the majority of FFAs had been released from larval adipocytes into the larval body cavity, thereby not being accounted for in the lipidomic analyses using the dissected fat body. Changes in different types of TGs, fatty acyl chains in TGs, and FFAs are shown in *SI Appendix*, Fig. S10. These observations show that overexpressing Lsd1 and Lsd2 substantially rescues defects in fat accumulation caused by active Wnt signaling, indicating a key role of Lsd-1 and Lsd-2 in mediating Wnt signaling induced lipid mobilization (Fig. 6*I*).

Interestingly, Lsd-1 is enriched on the surface of larger lipid droplets, whereas Lsd-2 tends to bind to smaller lipid droplets and can also be found in the cytoplasm (54). Consistent with previous reports that reducing Lsd-1 leads to larger LDs (53, 54), depletion of *Lsd-1* resulted in significantly larger but fewer LDs per adipocyte (Fig. 6*K* cf. Fig. 6*J*; quantified in Fig. 6*N*), accompanied by reduced TG accumulation (Fig. 6*O*). These observations support the idea that LDs with reduced Lsd-1 are more prone to fusion, forming larger LDs and favoring lipolysis. In contrast, depleting *Lsd-2* alone, using two different transgenic RNAi lines, did not significantly alter the size of LDs (*SI Appendix*, Fig. S9 *G*–*I*). Given that Wnt signaling negatively regulates the expression of Lsd-1 (Figs. 3*J* and 4), we asked whether inhibiting Wnt signaling could rescue the enlarged LD phenotype caused by *Lsd1* depletion (Fig. 6*I*). Indeed, the concurrent depletion of *arm/β-catenin* and *Lsd-1* in the larval adipocytes significantly rescued the enlarged LD phenotype induced by *Lsd-1* depletion (Fig. 6*M* cf. Fig. 6*K*; quantified in Fig. 6*N*). Depletion of Lsd-1 reduced the TG levels, which were also rescued by codepletion of *arm* (Fig. 6*O*). These observations support the notion that Wnt signaling negatively regulates *Lsd-1* transcription.

While the data presented in Figs. 3–6 could be explained by various models, the most parsimonious explanation is that active Wnt signaling stimulates lipolysis and lipid mobilization by repressing the expression of LDAPs such as *Lsd-1* and *Lsd-2* (Fig. 6*I*). We note that depleting Arm/β-catenin alone did not significantly affect LD size and TG levels (Fig. 6 *L*, *N*, and *O*) in this experiment. Several factors may contribute to this weak effect: First, the levels of TG were quantified using a kit that measures the glycerol concentration after the conversion of TGs, diacylglycerides, and monoacylglycerides into FFAs and glycerol; thus, the sensitivity of this method might be insufficient for accurately gauging the effects in this experiment. Second, constraints within the experimental timeframe during the short larval stage. This notion is supported by the observation that depleted Arm/β-catenin in adult flies significantly increased the TG levels (*SI Appendix*, Fig. S2 *D* and *E*). It is also important to note that the size of LDs does not necessarily correlate with the TG levels, underscoring the need for multiple complementary approaches to analyze lipid metabolism due to the intrinsic strengths and limitations of each method.

### Wnt Signaling Directly Represses the Transcription of Key Lipid Metabolism-Related Genes.

To determine whether Wnt signaling directly or indirectly regulates the transcription of genes related to lipid metabolism, we aimed to investigate the binding of dTCF to their promoters. However, a substantial obstacle was the scarcity of ChIP-grade antibodies for crucial components of the Wnt signaling pathway, as previously noted (9). To surmount this limitation, we leveraged CRISPR-Cas9 gene editing to insert an EGFP tag at the endogenous *dTCF/pan* locus (*SI Appendix*, Fig. S11*A*). This strategy led to the creation of the *dTCF^EGFP^* line, which was validated through genomic DNA sequencing (*SI Appendix*, Fig. S11 *B* and *C*). Additionally, we confirmed the nuclear location of the dTCF^EGFP^ protein in different larval tissues (*SI Appendix*, Fig. S11 *D* and *E*). Notably, *dTCF^EGFP^* homozygotes are fully viable and fertile, confirming that the presence of the EGFP tag does not impair the normal function of dTCF.

To map genome-wide dTCF-binding sites, we dissected larval adipocytes from third-instar *dTCF^EGFP^* homozygous larvae and conducted the cleavage under targets & release using nuclease (CUT&RUN) assay. While this technique offers efficiency and reproducibility advantages over conventional ChIP-Seq analyses (55–57), we encountered difficulties due to the large size of larval adipocytes and the presence of numerous lipid droplets, impeding DNA fragment release for sequencing. To address this, we attempted to purify nuclei from larval adipocytes before the CUT&RUN assay, which was hindered by the low yield due to abundant lipid droplets and a limited number of larval adipocytes (17). Despite these challenges, we generated and sequenced DNA libraries, revealing dTCF binding peaks in genes such as *Hnf4*, *FASN1*, *AcCoAS*, *Mondo*, *Lsd1*, *Pex13, Sccpdh1,* and *Sccpdh2*, along with several known Wnt signaling-related genes such as *fz2*, *fz3*, *fz4*, and *nkd* (*SI Appendix*, Fig. S12). However, the signal-to-noise ratio was suboptimal (*SI Appendix*, Fig. S12).

**Fig. 7.**
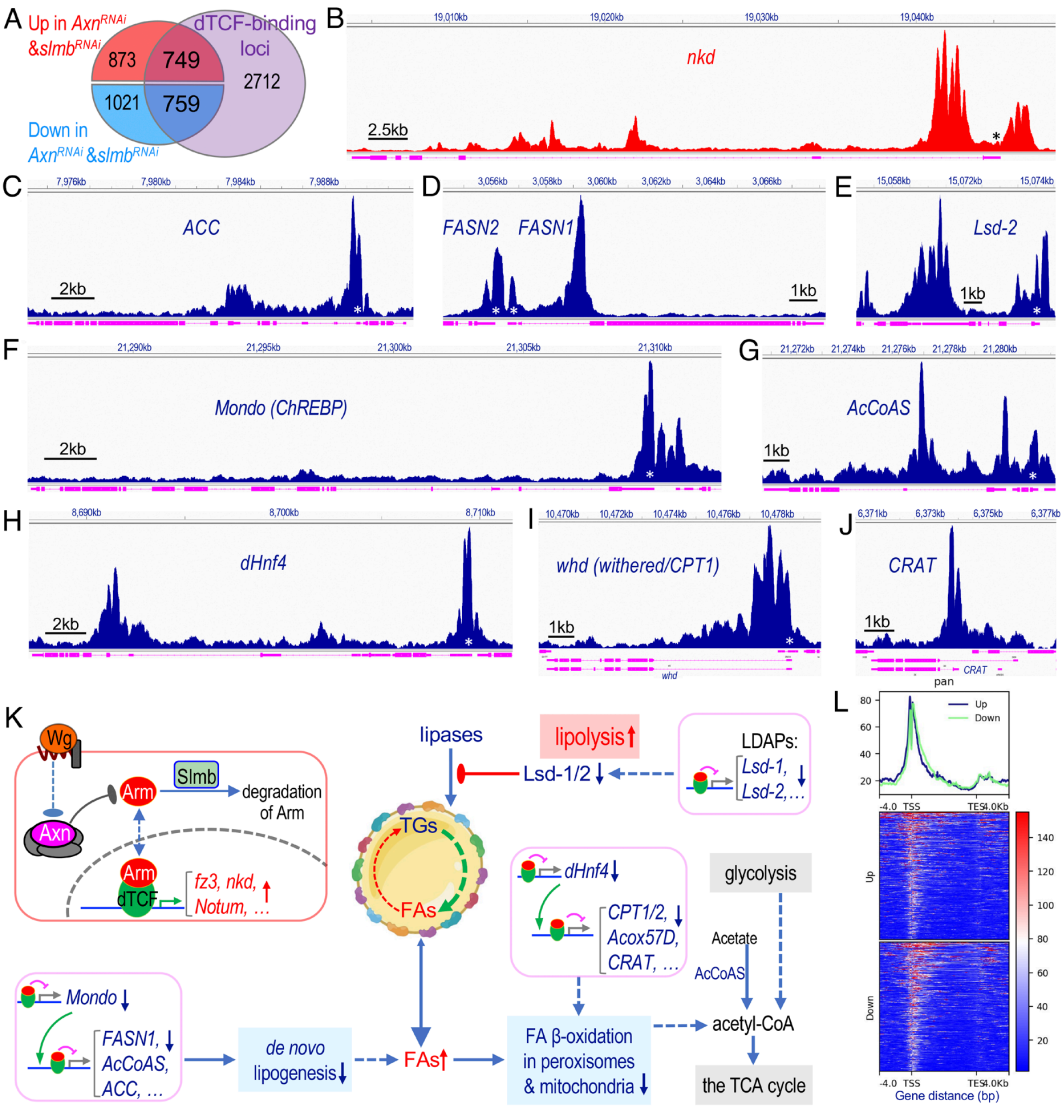
Direct repression of lipid-related genes by Wnt signaling. (*A*) Venn diagram showing genes altered in *Axn^RNAi^* or *slmb^RNAi^* fat body compared to loci bound by dTCF. (*B*–*H*) Genomic tracks displaying dTCF binding peaks at specific loci in wing discs: *nkd* (*B*), *ACC* (*C*), *FASN1* (*D*), *Lsd-2* (*E*), *Mondo* (*F*), *AcCoAS* (*G*), and *Hnf4* (*H*), *CPT1* (*withered*/*whd*; *I*), and *CRAT* (*J*). Visualized using the Integrative Genomics Viewer (IGV) browser with y-axis autoscaled, gene isoforms collapsed and shown in magenta. Genes activated by Wnt signaling are in red; down-regulated genes in blue. TSS are marked with an asterisk (*). (*K*) Schematic model showing Wnt signaling’s role in reducing TG accumulation and increasing FAs in adipocytes. Active Wnt signaling directly represses transcription of key lipogenesis (e.g., Mondo, FASN1, AcCoAS, and ACC), lipolysis (e.g., LDAPs including Lsd-1, Lsd-2, Sturkopf, and Spartin), and FAO (e.g., Hnf4, CPT1, CPT2, CRAT, and Acox57D) factors. LDAPs hinder lipase access to TGs inside lipid droplets. (*L*) Graph showing footprint and profile of dTCF binding motif among Wnt-activated and Wnt-repressed target genes. dTCF is enriched around the TSSs in both groups.

To overcome technical difficulties, we performed the CUT&RUN assay using wing discs from third-instar *dTCF^EGFP^* homozygous larvae. This approach identified 16,726 dTCF-binding peaks across 4,220 specific genetic loci (Fig. 7*A*), yielding superior data quality than purified adipocyte nuclei (*SI Appendix*, Fig. S12). Further examination of this gene list, compared with the genes identified through RNA-seq analyses using the *Axn^RNAi^* or *slmb^RNAi^* adipocytes, revealed 749 up-regulated and 759 down-regulated genes with dTCF-binding sites (Fig. 7*A*). Consensus motif enrichment analyses of the called dTCF-binding peaks within up-regulated genes in both *Axn^RNAi^* or *slmb^RNAi^* adipocytes revealed the presence of the AATCAAATCAAT motif, with a reverse complement sequence of ATTGATTTGATT (*SI Appendix*, Fig. S13*A*). Further analyses using the Tomtom Motif Comparison Tool identified a dTCF-binding motif within this sequence (*SI Appendix*, Fig. S13*A*). Similarly, analysis of all called dTCF-binding peaks in genes altered in both *Axn^RNAi^* or *slmb^RNAi^* adipocytes led to the identification of an AAATCAAAT motif, with a reverse complement sequence containing a TTTGAT motif (*SI Appendix*, Fig. S13*B*). This TTTGAT motif resembles the reported TCF (*SI Appendix*, Fig. S13*C*) or LEF1 binding motifs (*SI Appendix*, Fig. S13*D*) (58, 59). Furthermore, pathway enrichment analyses of the altered genes with called dTCF-binding peaks in both *Axn^RNAi^* or *slmb^RNAi^* adipocytes revealed the Wnt signaling pathway and several lipid metabolism-related pathways (*SI Appendix*, Fig. S14). These findings support the specificity of our CUT&RUN assay.

Notably, dTCF binding sites were found in established Wnt target genes such as *nkd* (Fig. 7*B*), *cycD*, *fz1*, *fz3*, *fz4*, *Notum*, and *Wnt4* (detailed in *SI Appendix*, Fig. S15*A*). Importantly, we observed multiple dTCF-binding peaks within genes encoding key lipogenic enzymes, including *ACC* (Fig. 7*C*) and *FASN1* (Fig. 7*D*), as well as other proteins involved in FA biosynthesis, such as *SREBP*, *Mondo* (Fig. 7*F*), *AcCoAS* (Fig. 7*G*), and others (*SI Appendix*, Fig. S15*B*). Given that the mRNA levels of these genes were reduced in the *Axn^RNAi^* and *slmb^RNAi^* adipocytes (Figs. 3 and 4), the identification of dTCF binding within the promoter regions of these key lipogenic genes suggests that Wnt signaling may directly repress their transcription.

The second class of genes displaying direct dTCF binding includes several LDAPs, such as *Lsd-2* (Fig. 7*E*), *Lsd-1*, *spartin*, *sturkopf*, and others (*SI Appendix*, Fig. S15*C*). Using a ChIP-qPCR assay, we observed significant enrichment of dTCF^EGFP^ on the promoters of *FASN1*, *Lsd-1*, and *nkd* (*SI Appendix*, Fig. S13 *E* and *F*), consistent with our CUT&RUN data. The third class of Wnt target genes includes *dHnf4* (Fig. 7*H*), *CPT1* (*withered*/*whd*; Fig. 7*I*), and *CRAT* (Fig. 7*J*), along with *CPT2*, *ScpX* (sterol carrier protein X-related thiolase), *Acox57D,* and other enzymes involved in FAO and electron transport chain (*SI Appendix*, Fig. S16*A*). CPT1, CPT2, and CRAT are crucial enzymes for transporting FAs into the mitochondria and peroxisomes (39–41). Furthermore, dTCF also binds to the promoters of several genes encoding peroxisomal proteins, such as *Pex1* (encoding Peroxin 1), *Pex6*, and *ACOX1* (encoding an acyl-CoA oxidase) (*SI Appendix*, Fig. S16*B*). Therefore, the collective increase in lipolysis, coupled with decreased lipogenesis and FAO, provides a straightforward explanation for the overall reduction of TGs and the concurrent rise in FFAs within adipocytes exhibiting active Wnt signaling (Fig. 7*K*).

To contrast the dTCF binding sites between Wnt-activated genes and Wnt-repressed genes, we aligned normalized counts from 82 up-regulated genes and 141 down-regulated genes, all of which had at least one enriched dTCF-binding peak within their gene span. Our analysis unveiled that dTCF is enriched around the transcription start sites (TSS), with no discernible difference observed between Wnt-activated versus Wnt-repressed target genes (Fig. 7*L*).

## Discussion

The mechanisms by which Wnt/Wg signaling modulates lipid homeostasis and represses the transcription of target genes have been poorly understood. This study elucidates how active Wnt signaling reduces TG levels by inhibiting de novo lipogenesis and promoting lipolysis. These coordinated actions result in decreased TG accumulation. Enhanced lipolysis, coupled with reduced FAO, leads to FFA accumulation in adipocytes. This interplay ultimately reduces fat accumulation in adipocytes with active Wnt signaling (Fig. 7*K*). Furthermore, this study suggests that active Wnt signaling directly represses the transcription of genes regulating lipogenesis, lipolysis, and FAO. These findings offer mechanistic insights into Wnt signaling-induced transcriptional repression in regulating lipid metabolism.

### Role of Wnt/Wg Signaling in Regulating Lipogenesis.

The inhibitory role of the canonical Wnt signaling pathway in mammalian adipogenesis is well established (12–14), but its regulation of lipogenesis, lipolysis, and FAO is less understood. In *Drosophila*, adipogenesis occurs late in embryogenesis, with adipocyte numbers remaining constant during the larval stage while their size increases due to lipid accumulation and nucleus endoreplication (15, 17, 18). This simplified process offers a unique opportunity to study Wnt signaling in lipogenesis and lipolysis in larval adipocytes. Due to the delayed effects of the Gal4-UAS system and transgenic RNAi approach in depleting *Axn* or *Slmb* in adipocytes, Wnt signaling activation likely occurs during the third instar larval stage, well after adipogenesis in the late embryonic stage. Additionally, our previous study revealed no obvious adipocyte defects in *Axin^127^* homozygous mutant larvae before mid-third instar (19). These observations suggest that Wnt signaling regulates lipid homeostasis independent of adipogenesis.

In adipocyte-specific β-catenin knockout mice and cultured mouse preadipocytes, lipogenic genes (*Srebf1*, *ACC1*, *FASN*, and *SCD1*) were reduced in β-cat deficient adipocytes (46). Overexpression of SREBP1c and ChREBP partially rescued *Scd1* and *Fasn* expression in β-Cat mutant adipocytes. ChIP-seq analysis showed Tcf7l2/TCF4 enrichment on lipogenic genes, suggesting Wnt signaling stimulates these lipogenic factors directly or indirectly through SREBP1c and ChREBP (46, 60). However, it is unclear whether Wnt signaling activation would similarly stimulate these genes. In juvenile turbot, GSK3 inhibitor LiCl treatment reduced *FASN1* expression in the liver and lowered plasma TG levels (47), aligning with Wnt signaling inhibiting *FASN1* transcription and lipogenesis. In a rat model, alcohol-induced fatty liver reduced nuclear β-Cat accumulation, both mitigated by a Wnt agonist (61), consistent with our findings.

A limitation in these studies, including ours, is the lack of precise quantification of Wnt signaling levels. While β-Cat loss disrupts basal Wnt signaling in mouse adipocytes (46), our study and others activate Wnt signaling by depleting *Axn* or *slmb* in *Drosophila*, using a GSK inhibitor in turbot, or a Wnt signaling agonist (47, 61). Varying Wnt signaling intensities may contribute to discrepancies in these studies regarding its role in lipogenesis. Future research with quantitative analyses and live imaging using sensitive Wnt activity reporters may clarify this issue.

### Wnt Signaling-Induced Lipid Mobilization in Different Cell Types.

This study primarily focused on larval adipocytes, raising questions about the generalizability of Wnt signaling’s effects on lipid mobilization and inhibition of lipogenesis in different cell types. We observed a significant reduction in fat accumulation in adult adipocytes with active Wnt signaling (*SI Appendix*, Fig. S2 *A*–*C*), while depleting either dTCF or Arm increased TG accumulation in adult adipocytes (*SI Appendix*, Fig. S2 *D* and *E*). This suggests that Wnt signaling affects lipid homeostasis beyond the larval stage. The regulatory mechanism of Wnt signaling on lipid metabolism may have cell-type-specific effects due to several factors.

First, the impact of Wnt signaling on lipid mobilization requires the presence of intracellular LDs, which vary in number, size, and lipid composition depending on cell types, physiological states, and pathological conditions (29). In *Drosophila*, LDs are mainly found in adipocytes, oenocytes, and parts of the gut (62). Cells like glia and larval salivary gland cells typically do not accumulate LDs (63–65), limiting their use in studying the effects of Wnt signaling on lipid mobilization. Wing disc cells contain small lipid droplets (66); Lsd-1 binds to larger lipid droplets and Lsd-2 binds to smaller lipid droplets (54). We observed low *Lsd-1* expression and Wnt signaling-mediated repression of *Lsd-2* in wing discs (Fig. 5*B* and *SI Appendix*, Fig. S6). The wing imaginal disc could be an interesting system to explore Lsd-2’s role in lipid metabolism.

Second, downregulation of Wnt signaling in the adult gut shows heterogeneous effects on lipid metabolism-related gene transcription (Fig. 5 *H* and *I* and *SI Appendix*, Fig. S8). Different cell types in the *Drosophila* gut exhibit distinct markers for stem cell renewal (67, 68). Our analyses (Fig. 5 *H* and *I*) focused on the anterior part of the R2 region within the adult midgut (69). Detailed characterization of specific cell types affected by Wnt signaling is needed to understand its role in regulating lipid metabolism-related genes.

Third, the effects of Wnt signaling on lipid metabolism-related gene transcription may vary across different tissues. For instance, unlike in adipocytes, the expression of *Lsd-1* and *Acox57D* in wing discs was very low (*SI Appendix*, Fig. S6). This discrepancy could be due to additional regulatory elements on the promoters of these genes, which integrate various developmental cues, including Wnt signaling. However, for genes expressed in both tissues, such as *Lsd-2* in wing discs, Wnt signaling represses their expression similarly to adipocytes (Figs. 4*B* and 5 *A* and *B*). Therefore, further investigation is necessary to understand how the mechanisms identified in this study intersect with other regulatory pathways in diverse developmental and physiological contexts.

### Physiological Relevance of Wnt Signaling-Regulated Lipid Metabolism.

While our primary focus was on investigating the impact of Wnt activation on lipid homeostasis, we also analyzed the effects of downregulation of Wnt signaling by depleting either Arm or dTCF/Pan on lipid metabolism in both larvae and adult flies, as well as the consequences of reducing Wnt signaling on the expression of lipid metabolism-related genes. We also observed that the expression level of *Lsd-2* is substantially lower along the D/V boundary (Fig. 5*A*), where endogenous Wnt/Wg signaling is active. Overall, the effects of Wnt signaling downregulation are consistent with our proposed model, where active Wnt signaling negatively regulates fat accumulation and represses the transcription of lipid metabolism-related genes. These findings underscore the physiological significance of Wnt signaling in regulating lipid homeostasis in larval and adult adipocytes.

However, the source of Wnt signal that regulates lipid homeostasis in adipocytes, and the environmental or internal cues to which it responds, remain unknown. Two possibilities merit consideration: First, Wnt ligands may originate from other tissues within the larval milieu. *Drosophila* has seven Wnt ligands, and it is unclear which one(s) play a physiological role in regulating lipid metabolism in larval adipocytes. According to Flybase, Wg is expressed in various tissues, including imaginal tissues, the nervous system, digestive system, reproductive system, muscle system, and several others during both larval and adult stages. Similarly, other Wnt ligands like Wnt2, Wnt4, Wnt5, Wnt6, Wnt10, and WntD exhibit either similar or more restricted expression patterns. It is conceivable that certain Wnt ligands diffuse into the body cavity, functioning as signaling molecules for interorgan communication. This hypothesis warrants future investigations.

Second, we cannot entirely exclude the potential crosstalk with other signaling pathways that regulate lipid homeostasis. Wnt signaling interacts with various other signaling pathways such as TGFβ (Decapentaplegic or Dpp in *Drosophila*), BMP (bone morphogenetic protein), Hedgehog, NF-κB, Notch, RTK (receptor tyrosine kinase), and Hippo signaling in different developmental contexts (70–72). While dTCF is considered the sole transcription factor downstream of the canonical Wnt signaling (8), it is plausible that other signaling pathways indirectly regulate Arm/dTCF activity, thereby modulating lipid homeostasis. This possibility also requires further exploration.

### Mechanism of Wnt Signaling-Induced Transcriptional Repression.

While much of the research on Wnt/Wg signaling focuses on gene activation, our study reveals an unexpected facet: Active Wnt/Wg signaling may directly repress the transcription of key lipid homeostasis regulators. Unlike the well-studied mechanisms of signaling-driven transactivation—activator insufficiency, cooperative activation, and default repression—signal-induced repression in major signaling pathways, including Wnt signaling, remains poorly understood in metazoans (11, 73). Fewer than 20 target genes repressed by active Wnt/Wg signaling have been characterized, including *dpp* in *Drosophila* leg discs (74), *CDH1* (E-cadherin) in mouse keratinocytes (75), and *stripe* in *Drosophila* embryos (76). Studies using *Drosophila* Kc cells identified additional repressed genes (8, 77). However, our study on larval adipocytes did not find these among dTCF targets, suggesting context-dependent transcriptional repression by Wnt/Wg signaling.

The exact mechanism of Wnt signaling-induced transcriptional repression remains unclear (9, 11, 78). Analysis of dTCF binding sequences in the *Ugt36Bc* locus has revealed that Wnt-repressed targets share the AGAWAW (W = A/T) consensus sequence, while Wnt-activated targets typically contain the SCTTTGWW (S = G/C) motif, potentially determining whether the Arm–dTCF complex acts as an activator or repressor (77). However, the prevalence of the AGAWAW motif throughout the *Drosophila* genome complicates the understanding of its role in repression (77). It is also unclear how dTCF alone discerns between activating and repressing genes. We postulate that dTCF combines with other DNA-bound transcription factors, likely repressors, to define Wnt-repressed targets, whereas Wnt-activated genes may lack binding sites for these repressors. Further research is needed to identify such repressors and elucidate how Wnt signaling triggers transcriptional repression, providing insights into the broader landscape of signaling-induced transcriptional repression.

### Implications of Wnt Signaling in Stimulating Lipolysis in Adipocytes.

This study revealed that Wnt signaling promotes lipolysis while inhibiting the β-oxidation of FAs in peroxisome and mitochondria, resulting in reduced TG levels and increased FFA levels (Fig. 7*K*). Our genetic and cell biological analyses on LDAPs, such as Lsd-1 and Lsd-2, support the notion that LDAPs on the surface of LDs regulate lipolysis by influencing the accessibility of lipases to TGs within LDs. Additionally, active Wnt signaling directly represses the transcription of genes encoding key LDAPs via dTCF, providing an explanation for its role in stimulating lipolysis and lipid mobilization. Consequently, active Wnt signaling leads to decreased TG accumulation and increased FFAs. These FFAs can be converted into phospholipids to form cellular membranes in proliferating cells for sustained cell division or released from adipocytes for use by other cells or tissues.

Aberrant Wnt signaling is prevalent in various human cancers, including colorectal, breast, and liver cancers (2, 6, 79, 80). We speculate that active Wnt signaling may also stimulate lipid mobilization in mammalian cells, promoting FFA and phospholipid production and providing growth advantages to cancer cells with hyperactive Wnt signaling. Interestingly, a recent study revealed mammary tumor cells can stimulate neighboring adipocytes to convert TGs into FFAs, likely by secreting yet unidentified factors (81). This potentially fuels the production of phospholipids and other membrane components necessary for cancer cell proliferation. In breast cancer, 12 out of 19 Wnt ligands are amplified, highly expressed, or activated by *TP53* mutation or TGF-β signaling (79). Thus, we speculate that breast cancer cells may exploit adjacent adipocytes for lipid mobilization, releasing FFAs through a Wnt signaling-dependent mechanism. If so, targeting enzymes involved in lipid catabolism, coupled with therapies like chemotherapy and immunotherapy, could offer synergistic advantages in cancer treatment.

### Limitations of the Study.

Despite the discussed limitations, there are opportunities for further enhancement in future research. While our model posits that Wnt signaling directly represses the transcription of key lipid-metabolism-related genes, supported by RNA-seq, CUT&RUN, HCR RNA-FISH, and developmental genetic analyses, a more detailed exploration of the molecular events underlying Wnt signaling-induced transcriptional repression is warranted. Additional validation of the CUT&RUN assay results and biochemical analyses are needed to confirm the effect of Wnt signaling on FAO in future studies.

## Materials and Methods

A detailed description of the *Materials and Methods* used in this study, including *Drosophila* stocks and maintenance, cell biological analyses, immunoblotting, the HCR RNA-FISH assay, cell culture and dual isotope radiolabeling experiments, RNA-seq sample preparation, library preparation, sequencing, differential gene expression analysis, gene ontology enrichment analysis, quantitative proteomics analysis, lipid sample preparation and shotgun lipidomics analysis, CRISPR Cas9 mediated tagging of dTCF with EGFP, CUT&RUN sequencing to identify the genome-wide dTCF-binding sites, the ChIP-qPCR assay, and statistical analyses, is provided in *SI Appendix*.

## Supplementary Material

Appendix 01 (PDF)

## Data Availability

The RNA-seq data and sequencing results from the CUT&RUN assay conducted in this study have been deposited in NCBI’s Gene Expression Omnibus and are accessible through GEO Series accession number GSE264356 (82).
