# Supplementary material for: Wnt/Wingless signaling promotes lipid mobilization through signal-induced transcriptional repression
Source: Proc Natl Acad Sci U S A. 2024 Jul 5;121(28):e2322066121. doi: 10.1073/pnas.2322066121 (PMC11252803; doi:10.1073/pnas.2322066121)
Supplement: Supplementary file 1 — Appendix 01 (PDF) [file pnas.2322066121.sapp.pdf]

Supplementary Information for

Wnt/Wingless signaling promotes lipid mobilization through signal-induced transcriptional repression

Mengmeng Liu[1]†, Rajitha-Udakara-Sampath Hemba-Waduge[1]†, Xiao Li[2], Xiahe Huang[3], Tzu-Hao Liu[1], Xianlin Han[4], Yingchun Wang[3], and Jun-Yuan Ji[1*]

[1] *Department of Biochemistry and Molecular Biology, Tulane University School of Medicine, Louisiana Cancer Research Center, 1700 Tulane Avenue, New Orleans, Louisiana 70112, USA.*

[2] *Lewis-Sigler Institute of Integrative Genomics, Princeton University, Princeton, New Jersey 08540, USA.*

[3] *State Key Laboratory of Molecular Developmental Biology, Department of Molecular Systems Biology, Institute of Genetics and Developmental Biology, Chinese Academy of Sciences, Beijing 100101, China.*

[4] *Barshop Institute for Longevity and Aging Studies, University of Texas Health Science Center at San Antonio, San Antonio, Texas 78229, USA.*

† These authors contributed equally to this work.

[*] *Corresponding author. Email: ji@tulane.edu*

This PDF file includes:

Supplementary Materials and Methods
SI References
Figure legends for Supplementary Figures S1 to S16
Figures S1 to S16
Tables S1 to S3

Supplementary Materials and Methods:

***Drosophila* stocks and maintenance:** The flies were maintained at 25˚C and fed with a standard diet comprising cornmeal, molasses, and yeast medium. Our analyses in this study focused exclusively on female larvae in the third instar wandering stage. Details about the specific strains and their genotypes are provided in Table S1. Arm^{EGFP} and Nkd^{EGFP} are intronic insertion lines with EGFP tags, generated using the MiMIC (Minos Mediated Integration Cassette) gene trap vector (1). The specific genotypes are '$Mi\{PT\text{-}GFSTF.1\}arm^{MI08675\text{-}GFSTF.1}$' and '$Mi\{PT\text{-}GFSTF.1\}nkd^{MI00209\text{-}GFSTF.1}/TM3,\ Sb^{1}\ Ser^{1}$', respectively.

Cell biological analyses: The methods for generating somatic clones in the larval fat body, dissection, and fixation of the larval fat body, as well as the protocols for immunostaining or staining with BODIPY, LipidTox, DAPI, and phalloidin, have been previously described (2, 3). Quantification of TG levels involved thin layer chromatography (TLC) and a TG quantification colorimetric kit, following established procedures as described earlier (2, 3). Additionally, for both S2R+ cell pellets or whole larvae, total lipids were extracted and applied to the TLC lanes. Lipid levels were normalized with respect to the total protein levels in each corresponding sample.

Immunoblotting: Cultured S2R+ cells were rinsed and suspended in ice-cold 1×PBS, followed by cell lysis in Tropix Lysis Solution (Applied Biosystems #2005117) containing a protease inhibitor cocktail (Roche #11836170001). This lysate was incubated on ice for 15 minutes, then subjected to centrifugation at 16,000×g for 30 min at 4°C. Protein concentrations were determined using Quick Start™ Bradford 1x Dye Reagent (Bio-Rad #5000205). For each sample, 20 µg of total protein was loaded into 10% Mini-PROTEAN TGX Gels (Bio-Rad #4561034) in SDS running buffer. The proteins were transferred onto nitrocellulose membranes using the Trans-Blot Turbo RTA Mini 0.2 µm Nitrocellulose Transfer Kit (Bio-Rad #1704270). The membranes were then incubated with a 1:1000 dilution of the anti-Arm primary antibody from the Developmental Studies Hybridoma Bank (DSHB Hybridoma Product N2 7A1 Armadillo, deposited by Eric Wieschaus), and a 1:2000 dilution of anti-Actin (Invitrogen #MA5-11869). A secondary antibody, anti-mouse-HRP-conjugated (Jackson ImmunoResearch), was used at a 1:5000 dilution. Visualization of the results was performed using the Western Lightning Plus-ECL (Perkin Elmer LLC #NEL105001EA).

The HCR RNA-FISH assay: The multiplexed in situ HCR RNA-FISH assay was performed as described previously (3), using specific probe sets and amplifiers obtained from Molecular Instruments. For probe sets targeting specific genes and their respective amplifiers, the following combinations were used : B1-Alexa Fluor 488 amplifiers with probe sets for *FASN1* (lot number PRQ415), *fz3* (RTD102), and *Lsd-1* (PRQ418); B2-Alexa Fluor 594 amplifiers with the probe sets for *AcCoAS* (lot number PRQ416) and *Lsd-2* (PRQ419); and B3-Alexa Fluor 647 amplifiers with probe sets for *ACC* (lot number PRQ417), *Acox57D-d* (PRQ420), *CRAT* (RTD104), and *Hnf4* (PRR571). Confocal images were captured using a Zeiss LSM900 confocal microscope system. Subsequently, the obtained images were processed using Adobe Photoshop 2021.

The cell culture and dual isotope radiolabeling experiments: *Drosophila* S2R+ cells (DGRC stock #150; https://dgrc.bio.indiana.edu//stock/150; RRID:CVCL_Z831) and S2-Tub-wg (DGRC stock #165; https://dgrc.bio.indiana.edu//stock/165; RRID:CVCL_1B57) were obtained from the *Drosophila* Genomics Resource Center (DGRC). The cells were cultured in Schneider's *Drosophila* Medium (Gibco #21720-024) supplemented with 10% FBS (ATCC #30-2020) and were maintained at 25°C. Wg-conditioned medium (WCM) and Control media (CM) were collected from S2-Tub-wg and S2R+ cells, respectively. Cells were seeded 24 h before collecting the supernatant. Supernatants were collected by centrifuging the cells at 500×g for 5 minutes and then filtered using 0.45 μm sterile syringe filters (Millex-HV #SLGV033RS).

For dual isotope radiolabeling experiments, S2R+ cells were seeded in six-well plates (2 ml/well; 3.5×10^6 cells/ml) for 24h. Radioactively labeled [^{14}C(U)]D-glucose (5 μl/well; 6.0×10^8 dpm/μmol) and [9,10-^{3}H(N)] Palmitic acid (2 μl/well; 1.05×10^{11} dpm/μmol) were added to the cells. Next, cells were treated with either WCM or CM. For WCM preparation, S2-Tub-wg cells were seeded 24 h before collecting the supernatant (5×10^6 cells/ml) by centrifuging. Control medium (CM) was prepared using S2R+ cells. S2R+ cells were collected from CM and WCM-treated wells at 0hr, 12hr, and 24hr. Cell pellets were washed with 1x PBS and TLC was performed as previously described (2, 4), with the following minor modifications. Specific regions corresponding to FFAs and TGs were scraped from the TLC plates and collected into scintillation vials. Counts per minute (cpm) values for ^{3}H and ^{14}C were determined by counting in 4 ml of CytoScint™ Liquid Scintillation Cocktail (Catalog number: 882453) in a Hidex 300 SL™ automatic liquid scintillation counter. Dpm (disintegrations per minute) values for ^{3}H and ^{14}C were computed using NIST (National Institute of Standards and Technology) traceable

standards. Efficiencies of ^3H and ^{14}C counting were 33.06% and 87.23% respectively for an optimized period of 100 seconds measuring time. Dpm values for ^{14}C and ^3H within TG and FFA were calculated using the respective efficiency figures. $^{14}C/^3H$ ratios were calculated, and normalized ratios were plotted. This protocol was used to perform dual isotope radiolabeling experiments to quantify TGs and FFAs in the cells over time to WCM treatment compared to the control.

RNA-seq sample preparation, library preparation, sequencing, differential gene expression analysis, and gene ontology enrichment analysis: Total RNA was extracted from dissected fat bodies of 20 third-instar larvae for each biological replicate. TRIzol Regent (Invitrogen) and the *Quick*-RNA MiniPrep kit (*Zymo Research*, R1055) were used for the RNA extraction and purification following the manufacturer's instructions. Ribosome-depleted stranded RNA libraries were prepared using the TruSeq Stranded Total RNA Library Prep kit (Illumina, 20020596) following to the manufacturer's manual. The prepared RNA libraries were sequenced by the BGI Americas Corporation. Sequencing reads were aligned to the Dm6 reference genome using the RNASTAR aligner (5). A count table was generated using featureCounts from Subread (6). Differential gene analysis within each group of experiments was performed by DESeq2 (7). Genes with adjusted p-value < 0.05 were considered differentially expressed. Gene ontology enrichment analysis was carried out using ClusterProfiler and DAVID GO (8, 9). The results of the gene ontology enrichment analysis were visualized using ggplot2 (10). Heatmaps were generated for visualization using the complexHeatmap package (11).

Quantitative proteomics analysis: Total proteins extracted from third instar whole larvae with the following genotypes: Axn^{127} homozygous mutant and w^{1118} (used as the control) were treated with 2.0 μM of BTZ mixed in fly food or DMSO (used as the control) (2). The samples were then analyzed using quantitative proteomics. Protein digestion was conducted using the filter-aided proteome preparation (FASP) with slight modifications (12). Briefly, proteins were reduced with 100 mM DTT at 37 °C for 1 h, and the lysates were transferred to the Microcon YM-30 centrifugal filter units (EMD Millipore Corporation, Billerica, MA). The lysis buffer was replaced twice with 200ul UA (8M Urea, 100mM Tris.Cl pH8.5). Alkylation was performed using 55 mM iodoacetamide (IAA, Sigma-Aldrich, Saint Louis, MO), the denaturing buffer was replaced with a buffer containing 0.1 M triethylammonium bicarbonate (TEAB, Sigma-Aldrich). Proteins were then digested using sequencing grade trypsin (Promega, Madison, WI) at 37 °C

overnight. The resulting tryptic peptides were labeled with acetonitrile-dissolved TMT reagents (Thermo Scientific, Rockford, IL). Equal amounts of labeled samples were mixed, and prefractionation was carried out using reversed-phase (RP)-high-performance liquid chromatography (HPLC). Peptides were fractionated on a phenomenex gemini-NX 5u C18 column (250 x 3.0 mm, 110 Å) (Torrance, CA, USA) employing a Waters e2695 separations HPLC system. The separated samples were collected and combined into 10 fractions. All fractions were dried using a Speed-Vac concentrator and subsequently desalted by StageTip (13). LC-MS/MS analysis was performed using an LTQ Orbitrap Elite mass spectrometer (Thermo Scientific) coupled online to an Easy-nLC 1000 in the data-dependent mode. Peptides were separated on a capillary analytic column (length: 25 cm, inner diameter: 75 µm) packed with C18 particles (diameter: 5 µm). The analysis was conducted in positive ion mode, and spectra were acquired within the mass range of 300-1800 m/z. Higher-energy collisional dissociation (HCD) was used to fragment the fifteen most intense ions from each MS scan. The raw MS files were processed through a database search using MaxQuant software (version 1.5). For this search, the *Drosophila melanogaster* proteome sequence database downloaded from uniprot (https://www.uniprot.org/) was used. The search parameters were configured as follows: type of search: MS2; protease used for protein digestion: trypsin; type of isobaric labels: 6-plex TMT; minimum score for unmodified peptides: 15; default values were applied for all other parameters.

Lipid sample preparation and shotgun lipidomics analysis: Larval fat bodies were dissected and promptly frozen using liquid nitrogen. Whole larvae samples were briefly rinsed in PBS and then frozen in liquid nitrogen. The frozen tissues were pulverized using a mortar and pestle pre-cooled with liquid nitrogen. The powdered samples were further homogenized by suspending them in a 10-fold diluted PBS using a Precellys Evolution® Homogenizer, running at 6000 rpm for 20 seconds, followed by 10-second pause. This homogenization cycle was repeated three times consecutively at 4 °C. Individual homogenates were subject to a protein assay to determine their protein content. An aliquot of each homogenate was then transferred to disposable glass test tubes. To enable quantification of various lipid classes, a mixture of lipid internal standards was added to each test tube, the quantities adjusted based on the tissue's protein content. Lipid extraction was performed by a modified Bligh and Dyer method, as previously described (14). Following extraction, all lipid extracts were flushed with N_2, capped, and stored at -20 ºC for future analysis.

For the ESI-MS analysis conducted after direct infusion, individual lipid extracts were further diluted to reach a final concentration of approximately 500 fmol/μL. The mass spectrometric analysis for lipids was conducted using a QqQ mass spectrometer (Thermo TSQ Altis, San Jose, CA). This instrument was equipped with an automated nanospray device (TriVersa NanoMate, Advion Bioscience Ltd., Ithaca, NY), which facilitated the ionization of lipid species through nano-ESI. This instrument was operated using Xcalibur software as previously described (15). To identify and quantify all lipid molecular species of interest, an in-house automated software program was used, adhering to the principles for quantification by mass spectrometry as elaborated previously (15). The fatty acyl chains of lipids were identified and quantified through neutral loss scans or precursor ion scans of corresponding acyl chains. These calculations were also performed using the same in-house software program (15). To ensure accurate quantification and comparisons, all lipid measurements were normalized to the protein content of the respective samples.

CRISPR Cas9 mediated tagging of dTCF/Pan with EGFP: *dTCF* gene structure information was retrieved from FlyBase (FBgn0085432) and primary amino acid sequence was retrieved from Uniprot (Q8IMA8). We analyzed the domain structure of the protein (https://www.ebi.ac.uk/interpro/result/InterProScan/iprscan5-R20220926-005559-0039-31654548-p1m/). Based on these analyses, we decided to introduce the EGFP tag at the C-terminus of the dTCF protein. Using the CRISPR Optimal target finder tool (http://targetfinder.flycrispr.neuro.brown.edu), two suitable sgRNAs target sites were strategically located within the last intron and 3'-UTR regions of the *dTCF* gene. The selected sgRNAs were then cloned into the pCFD3 vector, as described by Port et al. (http://www.crisprflydesign.org/plasmids/) (16). Primer design for subsequent steps was accomplished using NEB Builder (https://nebuilder.neb.com/#!/), and primer sequences are provided in Table S2. For actual gene editing and tagging, we employed the NEBuilder HiFi DNA Assembly Cloning Kit (NEB #E5520). This kit facilitated the introduction of both upstream and downstream homology arms, each spanning 1000bp each, as well as the last intron-exon regions and the EGFP coding region into a donor vector. The BAC clone 'BACR21B19', obtained from the BACPAC Resources Center, was used as the template for PCR to generate necessary DNA fragments. The donor vector and two sgRNA constructs in PCDF3 were injected into embryos, and the screening of the transgenic flies carrying endogenous EGFP tag was

carried out using classical fly genetic techniques. Additionally, PCR validation using genomic DNA extraction and sequencing was conducted to verify the successful integration of the EGFP tag.

CUT&RUN sequencing to identify the genome-wide dTCF-binding sites: For each sample, 20 wing discs from third-instar wandering larvae were dissected in Schneider medium. The wing discs were then transferred into Eppendorf tubes containing 100 μL 1x Wash buffer (from the CUT&RUN assay kit #86652, CST). We added 10 μL of activated Concanavalin A Magnetic bead suspension and allowed the tube to rotate at room temperature for 10 min. The Eppendorf tubes were placed on a Magnetic rack, the wash buffer was removed, and the beads were resuspended by adding 100 μL DBE buffer (provided in the kit). This resuspension step was conducted for 10 min at room temperature. We added 100 μL Antibody binding buffer containing the appropriate amount of primary antibody (anti-GFP, ab290) or negative control (rabbit mAb IgG from the kit). These tubes were placed on a nutator and incubated overnight at 4 °C. The subsequent steps were carried out by following the manufacturer's instructions as provided in the kit. The extracted DNA was purified by using DNA purification buffers and spin columns (#14209S, CST). DNA libraries were constructed using the TruSeq ChIP SMP Prep kit (IP-202-1012, 15034288, Illumina), following the sample preparation guide.

The sequencing data underwent processing using nf-core/cutandrun v2.0, an integrated pipeline designed for the CUT&RUN assay (17). The alignments from the pipeline underwent additional processing for peak calling with a significance threshold of -q 0.0001 and subsequent normalization using MACS3, which can be accessed at https://macs3-project.github.io/MACS/. For anti-GFP samples, the alignments were provided through the "-t" option, while control (IgG) samples utilized the "-c" option. To visualize the results in IGV (Integrative Genomics Viewer) with the *Drosophila* genome (assembly Release dm6) as the reference, we transformed the averaged and normalized pileup bedgraph file, which was generated alongside the peak calling step through MACS3 "-B" option, into a bigwig file. This conversion was accomplished using the bedGraphicToBigWig tool from the UCSC toolkit. The analysis of dTCF binding profiles was conducted using deepTools. To identify motifs within dTCF-binding sites, we utilized the narrow peaks identified from above MACS3 output. These peaks were then intersected with the regions ±2000bp around genes showing alterations in both Axn^{RNAi} and $slmb^{RNAi}$ RNA-seq data, by the intersect function from bedtools (18). The intersected peaks sequences were obtained by

getfasta function from bedtools. For *de novo* motif discovery, we input these sequences to the xstreme function from the MEME-Suite package with default settings (19).

Chromatin immunoprecipitation quantitative PCR (ChIP-qPCR) analyses: ChIP analyses were performed using dTCF/Pan^{EGFP} or w^{1118} (control) embryos collected over 24 hours, following a published protocol (20) with minor adjustments. After fixation and quenching of cross-linking, chromatin was fragmented to sizes ranging from 100 to 300 bp using a Covaris S220 Focused Ultrasonicator. The fragmented chromatin was then incubated overnight at 4˚C with GFP-Trap coupled to magnetic agarose beads (Bulldog, GTMA-020), while binding control magnetic agarose (Bulldog, BMAB-020) was used as a negative control. After washing, the chromatin was eluted from the beads, and the DNA was decrosslinked from protein. Finally, qPCR was performed using the purified DNA as the template, with specific primers listed in Table S3.

Statistical analyses: P-values in this study were computed using one-tailed unpaired *t*-tests, and error bars displayed represent the standard deviation. Each experiment in this work was subjected to analysis with a minimum of three independent biological replicates. */# for p<0.05, **/## for p<0.01, and ***/### for p<0.001, all determined through one-tailed unpaired *t*-tests.

SI References:

1. S. Nagarkar-Jaiswal *et al.*, A library of MiMICs allows tagging of genes and reversible, spatial and temporal knockdown of proteins in Drosophila. *Elife* **4** (2015).
2. T. Zhang *et al.*, Reversal of hyperactive Wnt signaling-dependent adipocyte defects by peptide boronic acids. *Proceedings of the National Academy of Sciences* **114**, E7469-E7478 (2017).
3. X. Li *et al.*, Cdk8 attenuates lipogenesis by inhibiting SREBP-dependent transcription in Drosophila. *Dis Model Mech* **15** (2022).
4. A. Hildebrandt, I. Bickmeyer, R. P. Kuhnlein, Reliable Drosophila body fat quantification by a coupled colorimetric assay. *PLoS One* **6**, e23796 (2011).
5. J. Widmann *et al.*, RNASTAR: an RNA STructural Alignment Repository that provides insight into the evolution of natural and artificial RNAs. *RNA* **18**, 1319-1327 (2012).

6. Y. Liao, G. K. Smyth, W. Shi, featureCounts: an efficient general purpose program for assigning sequence reads to genomic features. *Bioinformatics* **30**, 923-930 (2014).
7. M. I. Love, W. Huber, S. Anders, Moderated estimation of fold change and dispersion for RNA-seq data with DESeq2. *Genome Biol* **15**, 550 (2014).
8. T. Wu *et al.*, clusterProfiler 4.0: A universal enrichment tool for interpreting omics data. *Innovation (Camb)* **2**, 100141 (2021).
9. B. T. Sherman *et al.*, DAVID: a web server for functional enrichment analysis and functional annotation of gene lists (2021 update). *Nucleic Acids Res* **50**, W216-221 (2022).
10. H. Wickham (2016) ggplot2 : Elegant Graphics for Data Analysis. in *Use R!,* (Springer International Publishing : Imprint: Springer,, Cham), pp 1 online resource (XVI, 260 pages 232 illustrations, 140 illustrations in color.
11. Z. Gu, Complex heatmap visualization. *iMeta* **1**, e43 (2022).
12. J. R. Wisniewski, A. Zougman, M. Mann, Combination of FASP and StageTip-based fractionation allows in-depth analysis of the hippocampal membrane proteome. *J Proteome Res* **8**, 5674-5678 (2009).
13. J. Rappsilber, M. Mann, Y. Ishihama, Protocol for micro-purification, enrichment, pre-fractionation and storage of peptides for proteomics using StageTips. *Nat Protoc* **2**, 1896-1906 (2007).
14. M. Wang, X. Han, Multidimensional mass spectrometry-based shotgun lipidomics. *Methods Mol. Biol.* **1198**, 203-220 (2014).
15. K. Yang, H. Cheng, R. W. Gross, X. Han, Automated lipid identification and quantification by multi-dimensional mass spectrometry-based shotgun lipidomics. *Anal. Chem.* **81**, 4356-4368 (2009).
16. F. Port, H. M. Chen, T. Lee, S. L. Bullock, Optimized CRISPR/Cas tools for efficient germline and somatic genome engineering in Drosophila. *Proceedings of the National Academy of Sciences of the United States of America* **111**, E2967-2976 (2014).
17. C. Cheshire, C. West (Analysis pipeline for CUT&RUN and CUT&TAG experiments that includes QC, support for spike-ins, IgG controls, peak calling and downstream analysis. (https://nf-co.re/cutandrun/2.0).

18. A. R. Quinlan, BEDTools: The Swiss-Army Tool for Genome Feature Analysis. *Curr Protoc Bioinformatics* **47**, 11 12 11-34 (2014).
19. C. E. Grant, Bailey, T.L., XSTREME: Comprehensive motif analysis of biological sequence datasets. *bioRxiv* (2021).
20. V. Loubiere, A. Delest, B. Schuettengruber, A. M. Martinez, G. Cavalli, Chromatin Immunoprecipitation Experiments from Whole Drosophila Embryos or Larval Imaginal Discs. *Bio Protoc* **7**, e2327 (2017).

Figure legends for Supplementary Figures S1 to S16:

Fig. S1. Effects of Wnt signaling on larval adipocytes. (A-C) Representative confocal images of larval adipocytes from indicated genotypes, stained with DAPI (blue), and EGFP-tagged Arm shown in green. Genotypes: (A) *arm^{EGFP}/+; SREBP-Gal4/+;* +; (B) *arm^{EGFP}/+; SREBP-Gal4/UAS-Axn^{RNAi};* +; and (C) *arm^{EGFP}/+; SREBP-Gal4/+; UAS-$slmb^{RNAi}$/+*. (D/E) Stimulation of Wnt target gene *nkd* expression by depleting Axn in larval adipocytes. Larval adipocytes were stained with Phall (red) and DAPI (blue). Genotypes: (D/D') *+; r4-Gal4/nkd^{EGFP}*; (E/E') *UAS-Axn^{RNAi}/+; r4-Gal4/nkd^{EGFP}*. Elevated Nkd^{EGFP} in small adipocytes compared to large adipocytes (indicated by asterisks *). (F-K) Depleting *Axn* using various Gal4 lines active in adipocytes resulted in similar lipid accumulation defects. Genotypes: (F) *+; SREBP-Gal4/+*; (G) *UAS-Axn^{RNAi}/+; SREBP-Gal4/+*; (H) *FB-Gal4/+; +*; (I) *FB-Gal4/UAS-Axn^{RNAi}; +*; (J) *+; r4-Gal4/+*; and (K) *UAS-Axn^{RNAi}/+; r4-Gal4/+*. (L) Quantification of TG levels in *Axn*-depleted larvae using multiple Gal4 drivers active in adipocytes, including *dCg-Gal4*, *SREBP-Gal4*, and *r4-Gal4* (n = 3 biological repeats). Results were normalized to control (*dCg-Gal4/+; +*). (M/N) Rescue of lipid accumulation defects by depleting dTCF in *Axn*-depleted larval adipocytes. Genotypes: (M) *UAS-$dTCF^{RNAi}$/+; r4-Gal4/+*; and (N) *UAS-$dTCF^{RNAi}$/UAS-Axn^{RNAi}; r4-Gal4/+*. The scale bars in panels C and G apply to all images in this figure: 20 µm.

Fig. S2. Effects of Wnt signaling on adult adipocytes. (A, B) Representative confocal images of adult adipocytes, stained with DAPI, Phall, and BODIPY. Genotypes: (A) *+; r4-Gal4/+*; and (B) *UAS-Axn^{RNAi}/+; r4-Gal4/+*. The scale bar in panel B corresponds to images A and B: 40 µm. (C) Quantification of the TG levels in female adults of indicated genotypes (n > 3 biological repeats). Results in (C) were normalized to control (*+; r4-Gal4/+*). (D/E) Quantification of the TG levels in 5-day-old (D) or 30-day-old (E) female adults of indicated genotypes (n > 3 biological repeats). Results were normalized to control (*+; dCg-Gal4/+*). Additional genotypes include: *dCg-Gal4/UAS-$dTCF^{RNAi}$; +* and *dCg-Gal4/UAS-$dTCF^{RNAi}$; +*.

Fig. S3. Quantitative lipidomics analyses of different types of TGs and FFAs. Whole larvae at the third instar wandering stage were used in this analysis. (A-D) Levels of different types of TGs; (E-F) Levels of different types of FFAs. Specific genotypes shown in (A) are color-coded. Asterisks (*) represent comparisons between '*dCg-Gal4/+*' and '*dCg-Gal4/UAS-Axn^{RNAi}*', while pound signs (#) indicate comparisons between '*dCg-Gal4/+*' and '*dCg/UAS-$dTCF^{RNAi}$*'. */#:

p<0.05; **/##: p<0.01; and ***/###: p<0.001, all based on one-tailed *t*-tests.

Fig. S4 Bioinformatic analyses of RNA-seq data for genes altered in *Axn*^{RNAi} or *slmb*^{RNAi} fat bodies. (A) Heatmap illustrating gene expression changes in fat bodies related to the 'Wnt signaling' pathway. (B, C) Pathway enrichment bubble plots displaying major pathways significantly altered in larval fat body upon depletion of either *Axn* (B) or *slmb* (C). Genotypes: (B) *SREBP-Gal4/UAS-Axn*^{RNAi}*;* +; and (C) *SREBP-Gal4/+; UAS-slmb*^{RNAi}/+. (D) Heatmap displaying downregulation of gene expression in the 'Biosynthesis of unsaturated fatty acids' pathway in *Axn*^{RNAi} or *slmb*^{RNAi} fat body. (E) Heatmap illustrating changes in gene expression for genes related to the 'Peroxisome' pathway.

Fig. S5. Detection of mRNA transcripts in larval adipocytes using the HCR assay. mRNA transcripts of key genes visualized within larval adipocytes: *Lsd-1/FASN1* (green), *Lsd-2/AcCoAS* (orange), and *Acox57D/ACC* (magenta). Large adipocytes, identified as "Wnt LO" (indicating low Wnt/Wg signaling), outlined with dotted lines. In contrast, adjacent smaller adipocytes are labeled as "Wnt HI" (representing high Wnt/Wg signaling). Genotypes: (A/D) *dCg-Gal4/+;* +; (B/E) *dCg-Gal4/UAS-Axn*^{RNAi}*;* +; and (C/F) *dCg-Gal4/+; UAS-slmb*^{RNAi}/+. Scale bars in panels C and F: 20 µm.

Fig. S6 Detection of mRNA transcripts in wing discs using the HCR assay. Confocal images displaying mRNA transcripts of *Lsd-2* (orange; A'-D'), *Lsd-1* (green; A"-D"), and *Acox57D* (red; A"'-D"') in the same discs as shown in Fig. 5A and Fig. 5B, respectively. (A-A"'/C-C"') Single focal plane; (B-B"'/D-D"') Z-stack projection; (a-d) BFP; merged images are shown in A-D. The dorsal/ventral (D/V) boundary and the anterior/posterior (A/P) boundaries are shown in C. Genotypes: (A/B) *en-Gal4/+; UAS-BFP/+*; and (C/D) *en-Gal4/UAS-Axn*^{RNAi}*; UAS-BFP/+*. Scale bar in panel (d): 20 µm.

Fig. S7 Physiological role of Wnt signaling in regulating lipid metabolism-related gene expression in larval adipocytes. (A-D) Merged confocal images showing mRNA transcripts of *FASN1* (green) and *Lsd-2* (magenta) in larval adipocytes of indicated genotypes using the HCR assay. Single-channel images: (A'-D'): *FASN1*; (A"-D") *Lsd-2*; and (A"'-D"') DAPI. Genotypes: (A) +*; r4-Gal4/*+; (B) +*; r4-Gal4/UAS-notum*$^{+}$; (C) +*; r4-Gal4/UAS-arm*^{RNAi}; and (D) *UAS-dTCF*^{RNAi}*/+; r4-Gal4/+*. Scale bar in panel D"': 10 µm.

Fig. S8 Detection of mRNA transcripts in adult midgut using the HCR assay. (A-C) Merged confocal images presenting mRNA transcripts of *FASN1* (green), *Lsd-2* (orange), and *CRAT*

(magenta) using the HCR assay in the R2 region of adult midgut of the following genotypes: (A) *+; r4-Gal4/+*; (B) *UAS-dTCF^{RNAi}/+; r4-Gal4/+*; (C) *UAS-dTCF^{DN}/+; r4-Gal4/+*. Nuclei are stained with DAPI (blue). The scale bar in panel C'": 5 µm.

Fig. S9 Confocal images of larval adipocytes from the indicated genotypes. (A-D) Larval fat bodies stained with DAPI (blue) and LipidTox (red). Genotypes: (A/A') *dCg-Gal4/+; UAS-Lsd1^{eGFP}/+*; (B/B') *dCg-Gal4/UAS-Axn^{RNAi}; UAS-Lsd1^{eGFP}/+*; (C/C') *dCg-Gal4/+; UAS-Lsd2^{eGFP}/+*; (D/D') *dCg-Gal4/UAS-Axn^{RNAi}; UAS-Lsd2^{eGFP}/+*; (E/E') *dCg-Gal4/+; UAS-Lsd1^{eGFP}/UAS- Lsd2^{eGFP}*; and (F/F') *dCg-Gal4/UAS-Axn^{RNAi}; UAS- Lsd1^{eGFP}/UAS- Lsd2^{eGFP}*. The scale bar in panel F' applies to images A-F: 20 µm. (G-I) Larval fat bodies stained with DAPI (blue), BODIPY (green), and Phall (red). Genotypes: (G) *+; SREBP-Gal4/+*; (H) *+; SREBP-Gal4/UAS-Lsd2^{RNAi}* (BL-34617); and (I) *+; SREBP-Gal4/UAS-Lsd2^{RNAi}* (BL-32846). The scale bar in panel G applies to images G-I: 10 µm.

Fig. S10. Quantitative lipidomics measurement in fat body from third instar larvae. These figures present additional results from quantitative lipidomics analysis, focusing on various types of lipids in fat body dissected from third instar larvae. (A/B) Triglyceride (TG) levels, (C) Amount of fatty acyl chains within the total TG pool, and (D) FFAs contents. Specific genotypes are color coded and shown in panel A. Asterisks (*) represent comparisons between '*dCg-Gal4/+; +*' and '*dCg-Gal4/UAS-Axn^{RNAi}; +*', while pound signs (#) indicate comparisons between '*dCg-Gal4/+; UAS-Lsd1^{eGFP}/UAS-Lsd2^{eGFP}*' and '*dCg/UAS-Axn^{RNAi}; UAS-Lsd1^{eGFP}/UAS-Lsd2^{eGFP}*'. Statistical significance: * or #: $p<0.05$; ** or ##: $p<0.01$; *** or ###: $p<0.001$; these significance values are based on one-tailed unpaired *t*-tests.

Fig. S11. Generation and validation of the *dTCF^{EGFP} Drosophila* strain. This figure outlines the process of creating and validating the *dTCF^{EGFP} Drosophila* strain. (A) The design of the donor template using the *pGEM-T-dTCF^{EGFP}* vector. (B) The validation of the *dTCF^{EGFP}* line by PCR using genomic DNA from *dTCF^{EGFP}* homozygous larvae. (C) Sequencing results confirming the presence and accuracy of the dTCF^{EGFP} in the genomic DNA. (D/D') Localization of dTCF in the nuclei of larval adipocytes. (E/E') Localization of dTCF in the nuclei of wing imaginal disc cells. (D'/E') Merged images with DAPI staining of nuclei (blue). The scale bar in panel D' applies to images D/D' and E/E': 10 µm.

Fig. S12. Visualization of dTCF binding at genomic loci in CUT&RUN assay data from wing discs and purified adipocyte nuclei samples. To facilitate comparison, the tracks are

color-coded as follows: blue tracks represent genes downregulated by Wnt signaling in the wing disc sample, red tracks indicate genes stimulated by Wnt signaling in the wing disc sample, and orange tracks depict data from the purified adipocyte nuclei sample. Arrows highlight overlapping peaks observed in both sample sources, with a noticeable increase in background noise peaks in the purified adipocyte nuclei sample. The y-axis is autoscaled, while multiple transcript isoforms are collapsed and presented in magenta beneath each respective track. Specific genes included are *Hnf4* (A), *FASN1* (B), *AcoCoAS* (C), *Mondo* (D), *Lsd-1* (E), *Pex13* (F), *Sccpdh1* (G), *Sccpdh2* (H), *fz2* (I), *fz3* (J), *fz4* (K), and *nkd* (L).

Fig. S13. Motif enrichment analyses of genes with called dTCF/Pan binding peaks. (A, B) Identification of a dTCF/Pan motif based on motif enrichment analysis of upregulated genes in both Axn^{RNAi} and $slmb^{RNAi}$ adipocytes (A), and all called peaks in genes altered in both Axn^{RNAi} and $slmb^{RNAi}$ adipocytes (B). For comparison, screenshots of motif bound by TCF HMG domain (C; from PMID 24516405) and LEF2 motif (D; from PMID 20696899) are included. (E, F) Validation of the CUT&RUN results using the ChIP-qPCR assay. (E) Relative enrichment of $dTCF^{EGFP}$ was assessed on the promoters of Wnt target gene *nkd*, and lipid metabolism-related genes, *FASN1* and *Lsd-1*. Samples were from $dTCF^{EGFP}$ homozygous embryos, with the IgG control shown in black. The primer pair '*nkd-1C*' was positioned away from the $dTCF^{EGFP}$-binding peaks ('*nkd-2C*') in the *nkd* promoter region. (F) Negative controls used w^{1118} embryos.

Fig. S14. Pathway enrichment analyses of genes altered in both Axn^{RNAi} and $slmb^{RNAi}$ adipocytes with called dTCF/Pan binding peaks. This analysis identified the Wnt signaling pathway and several pathways related to lipid metabolism, which are highlighted.

Fig. S15. dTCF/Pan binding at diverse genomic loci. This figure provides a visual representation of dTCF binding at various genomic loci, organized by their functional pathways. Genes activated by Wnt signaling are shown in red tracks, while genes downregulated by Wnt signaling are displayed in blue tracks. (A) Genes related to the Wnt signaling pathway. (B) Genes related to fatty acid biosynthesis pathway. (C) Genes related to LDAPs and lipid droplets. The asterisk (*) indicates the transcription start site (TSS). The y-axis is autoscaled, while different transcript isoforms are consolidated and displayed in magenta beneath each respective track.

Fig. S16. dTCF/Pan binding at diverse genomic loci. (A) Genes related to FAO and the electron transport chain. (B) Genes related to peroxisome. Genes downregulated by Wnt

signaling are displayed in blue tracks. The y-axis is autoscaled, while different transcript isoforms are consolidated and displayed in magenta beneath each respective track.

Supplementary Tables S1 to S3:

Table S1 List of the *Drosophila* stocks used in this study.

Table S2 Primers used to generate the *dTCF*^{EGFP} strain via CRISPR-Cas9.

Table S3 Primers used for the ChIP-qPCR assay.

Figures S1-S16

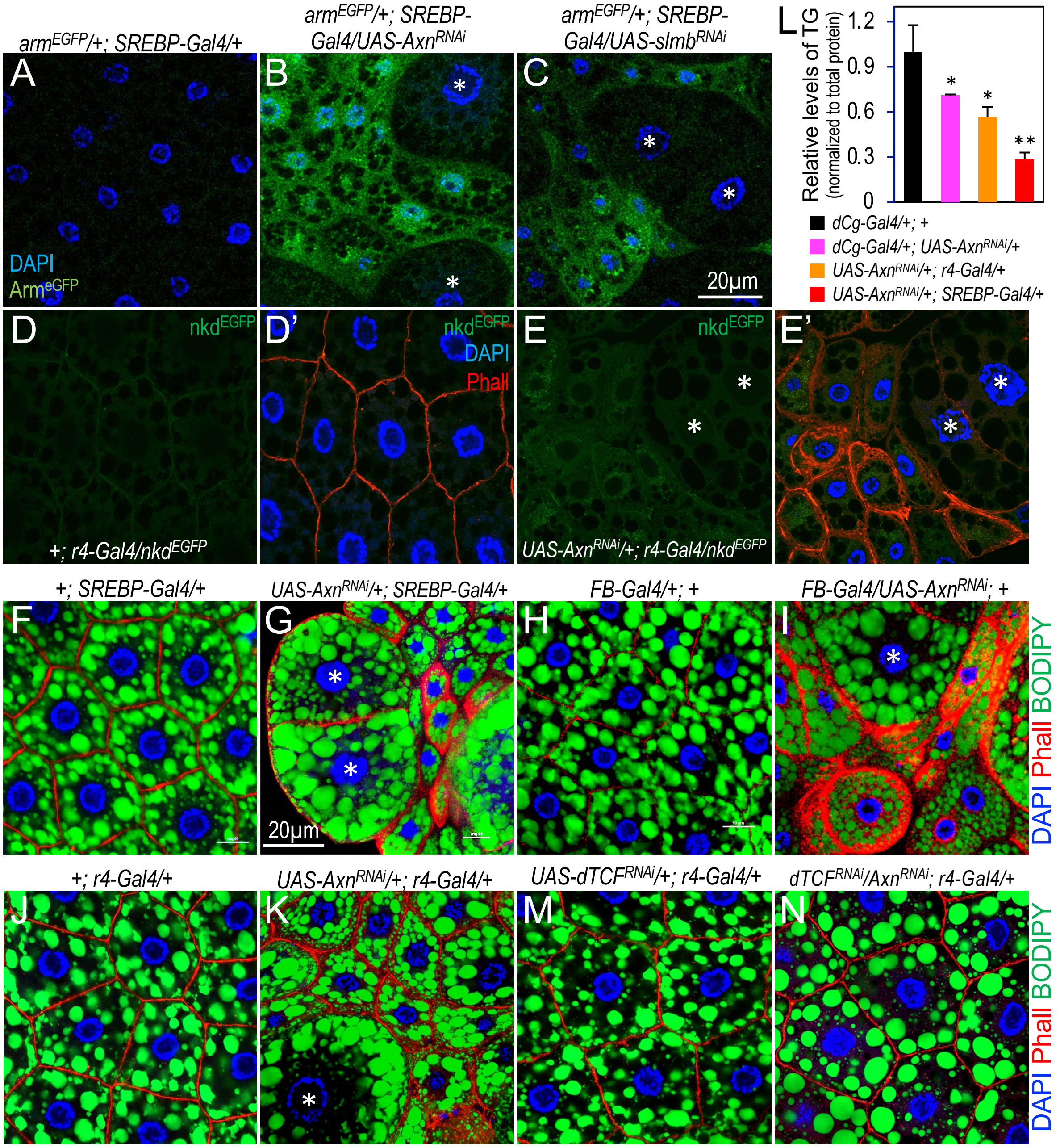

Fig. S1. Effects of Wnt signaling on larval adipocytes. (A-C) Representative confocal images of larval adipocytes from indicated genotypes, stained with DAPI (blue), and EGFP-tagged Arm shown in green. Genotypes: (A) *arm*^{EGFP}*/+; SREBP-Gal4/+;* +; (B) *arm*^{EGFP}*/+; SREBP-Gal4/UAS-Axn*^{RNAi}*;* +; and (C) *arm*^{EGFP}*/+; SREBP-Gal4/+; UAS-slmb*^{RNAi}*/+*. (D/E) Stimulation of Wnt target gene *nkd* expression by depleting Axn in larval adipocytes. Larval adipocytes were stained with Phall (red) and DAPI (blue). Genotypes: (D/D') +*; r4-Gal4/nkd*^{EGFP}; (E/E') *UAS-Axn*^{RNAi}*/+; r4-Gal4/nkd*^{EGFP}. Elevated Nkd^{EGFP} in small adipocytes compared to large adipocytes (indicated by asterisks *). (F-K) Depleting *Axn* using various Gal4 lines active in adipocytes resulted in similar lipid accumulation defects. Genotypes: (F) +*; SREBP-Gal4/*+; (G) *UAS-Axn*^{RNAi}*/+; SREBP-Gal4/*+; (H) *FB-Gal4/+;* +; (I) *FB-Gal4/UAS-Axn*^{RNAi}*;* +; (J) +*; r4-Gal4/*+; and (K) *UAS-Axn*^{RNAi}*/+; r4-Gal4/+*. (L) Quantification of TG levels in *Axn*-depleted larvae using multiple Gal4 drivers active in adipocytes, including *dCg-Gal4*, *SREBP-Gal4*, and *r4-Gal4* (n = 3 biological repeats). Results were normalized to control (*dCg-Gal4/+;* +). (M/N) Rescue of lipid accumulation defects by depleting dTCF in *Axn*-depleted larval adipocytes. Genotypes: (M) *UAS-dTCF*^{RNAi}*/+; r4-Gal4/*+; and (N) *UAS-dTCF*^{RNAi}*/UAS-Axn*^{RNAi}*; r4-Gal4/*+. The scale bars in panels C and G apply to all images in this figure: 20 μm.

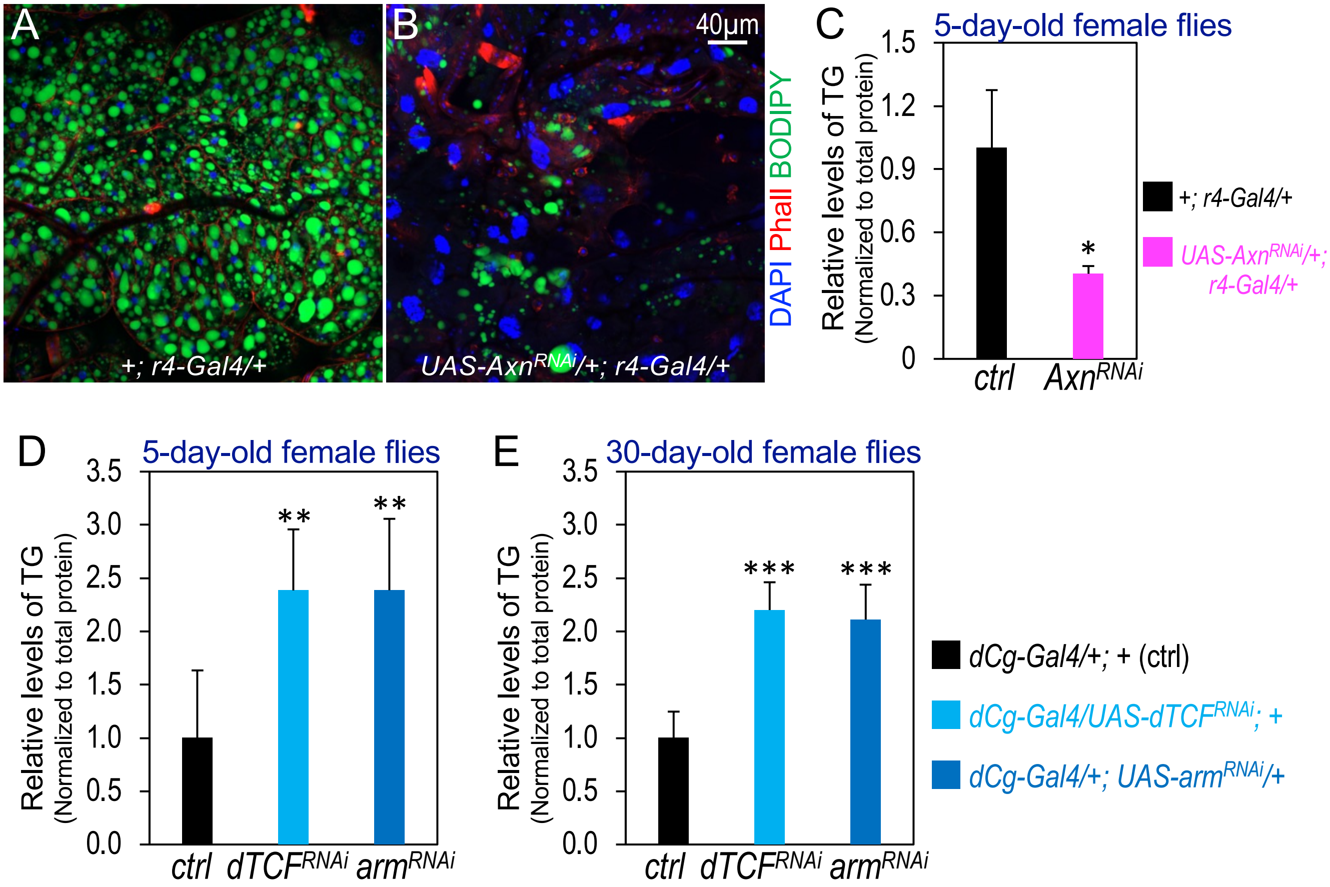

Fig. S2. Effects of Wnt signaling on adult adipocytes. (A, B) Representative confocal images of adult adipocytes, stained with DAPI, Phall, and BODIPY. Genotypes: (A) +*; r4-Gal4/*+; and (B) *UAS-Axn^{RNAi}/+; r4-Gal4/+*. The scale bar in panel B corresponds to images A and B: 40 µm. (C) Quantification of the TG levels in female adults of indicated genotypes (n > 3 biological repeats). Results in (C) were normalized to control (+*; r4-Gal4/*+). (D/E) Quantification of the TG levels in 5-day-old (D) or 30-day-old (E) female adults of indicated genotypes (n > 3 biological repeats). Results were normalized to control (+*; dCg-Gal4/*+). Additional genotypes include: *dCg-Gal4/UAS-dTCF^{RNAi}; +* and *dCg-Gal4/UAS-dTCF^{RNAi}; +*.

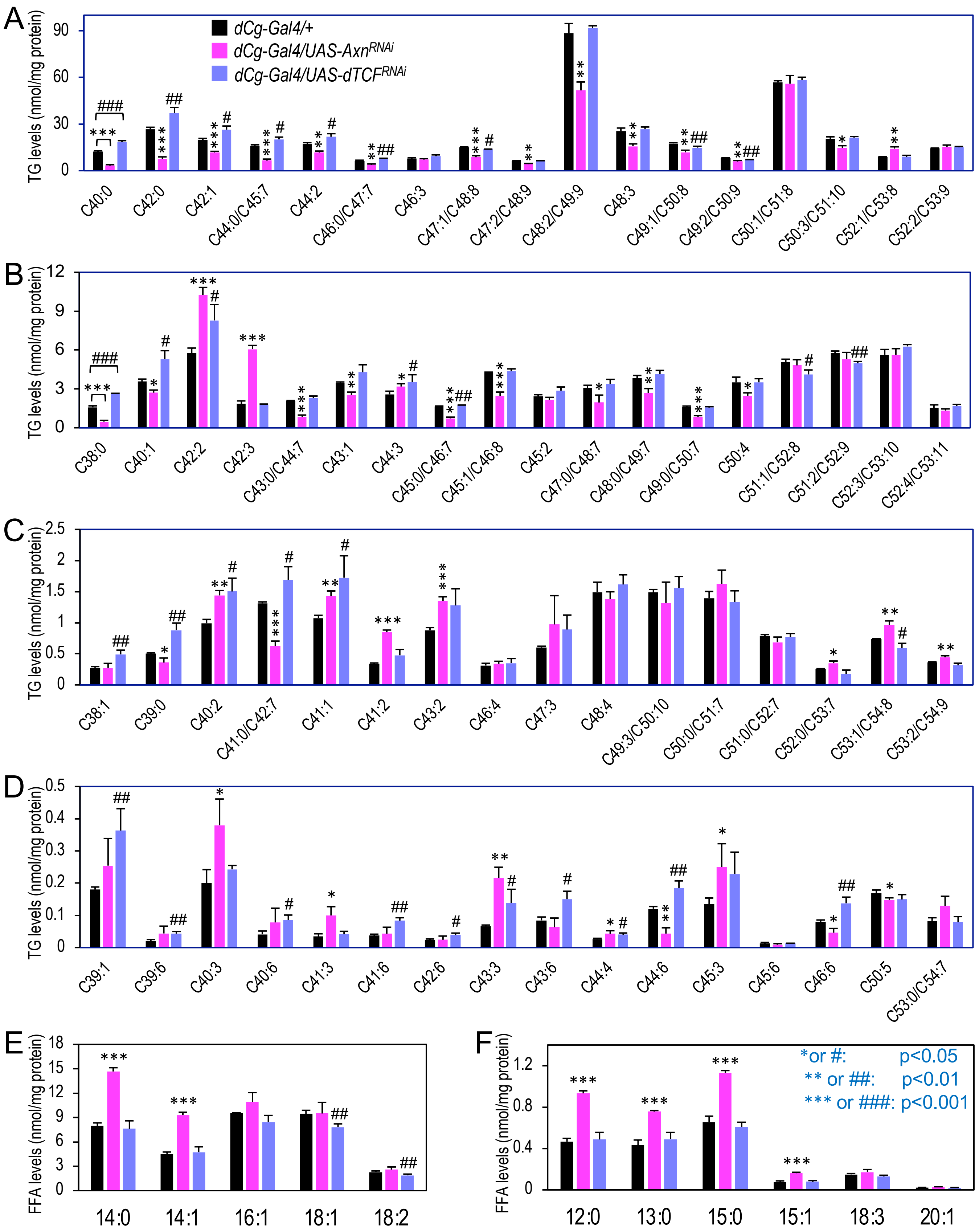

Fig. S3. Quantitative lipidomics analyses of different types of TGs and FFAs. Whole larvae at the third instar wandering stage were used in this analysis. (A-D) Levels of different types of TGs; (E-F) Levels of different types of FFAs. Specific genotypes shown in (A) are color-coded. Asterisks (*) represent comparisons between '*dCg-Gal4/+*' and '*dCg-Gal4/UAS-Axn*^{RNAi}', while pound signs (#) indicate comparisons between '*dCg-Gal4/+*' and '*dCg/UAS-dTCF*^{RNAi}'. */#: p<0.05; **/##: p<0.01; and ***/###: p<0.001, all based on one-tailed *t*-tests.

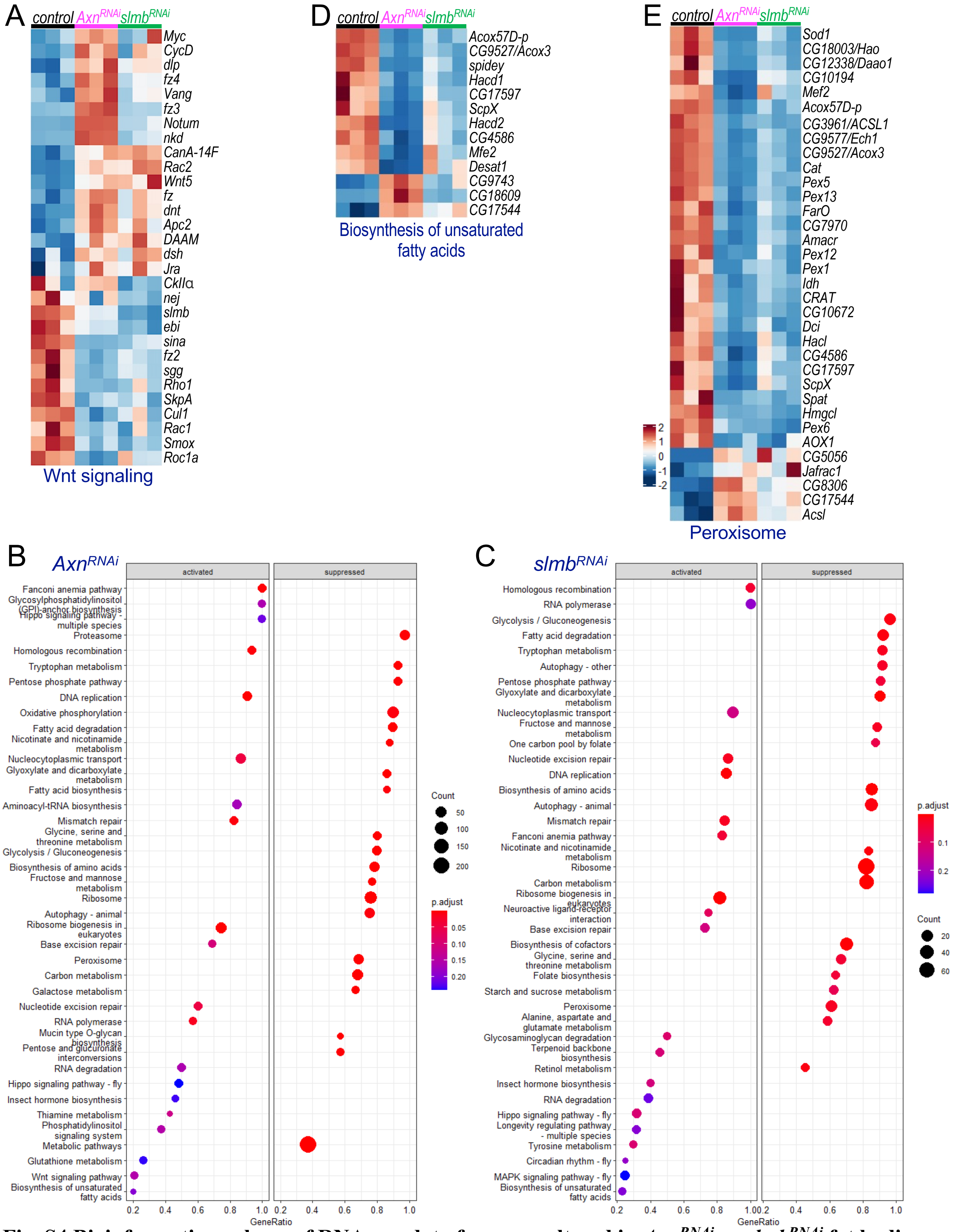

Fig. S4 Bioinformatic analyses of RNA-seq data for genes altered in *Axn*^{RNAi} or *slmb*^{RNAi} fat bodies. (A) Heatmap illustrating gene expression changes in fat bodies related to the 'Wnt signaling' pathway. (B, C) Pathway enrichment bubble plots displaying major pathways significantly altered in larval fat body upon depletion of either *Axn* (B) or *slmb* (C). Genotypes: (B) *SREBP-Gal4/UAS-Axn*^{RNAi}*; +;* and (C) *SREBP-Gal4/+; UAS-slmb*^{RNAi}*/+*. (D) Heatmap displaying downregulation of gene expression in the 'Biosynthesis of unsaturated fatty acids' pathway in *Axn*^{RNAi} or *slmb*^{RNAi} fat body. (E) Heatmap illustrating changes in gene expression for genes related to the 'Peroxisome' pathway.

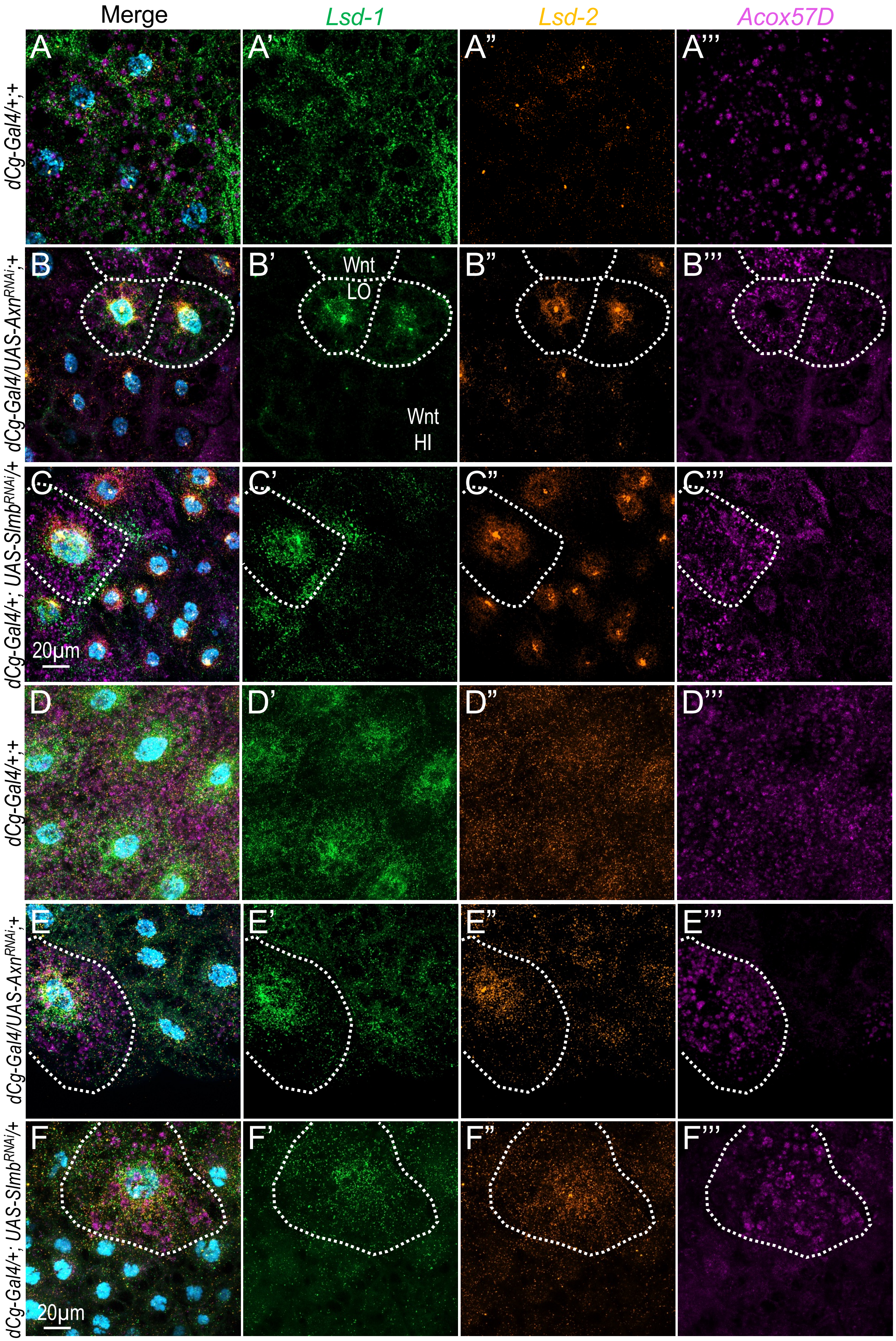

Fig. S5. Detection of mRNA transcripts in larval adipocytes using the HCR assay. mRNA transcripts of key genes visualized within larval adipocytes: *Lsd-1/FASN1* (green), *Lsd-2/AcCoAS* (orange), and *Acox57D/ACC* (magenta). Large adipocytes, identified as "Wnt LO" (indicating low Wnt/Wg signaling), outlined with dotted lines. In contrast, adjacent smaller adipocytes are labeled as "Wnt HI" (representing high Wnt/Wg signaling). Genotypes: (A/D) *dCg-Gal4/+;* +; (B/E) *dCg-Gal4/UAS-Axn*^{RNAi}*;* +; and (C/F) *dCg-Gal4/+; UAS-slmb*^{RNAi}/+. Scale bars in panels C and F: 20 µm.

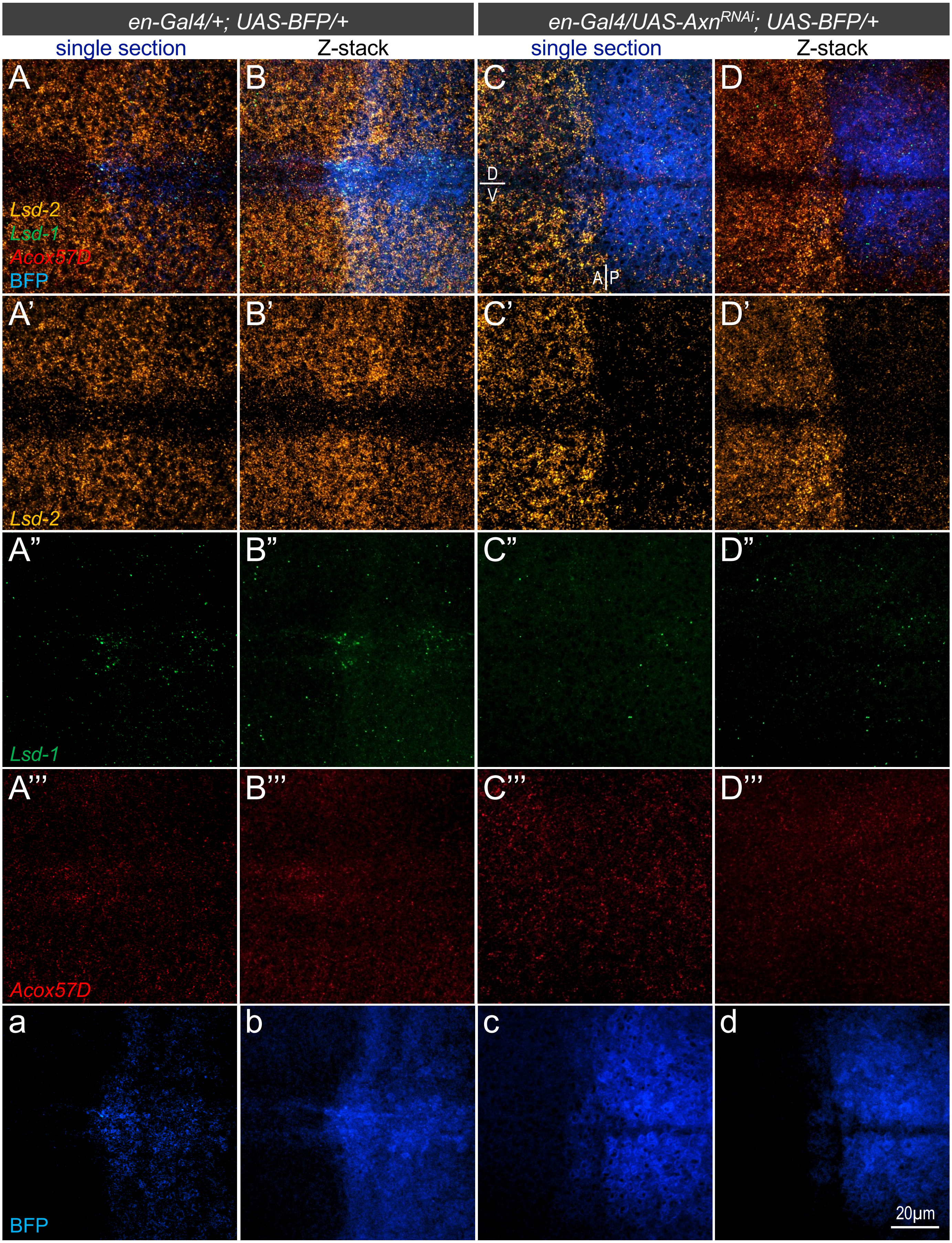

Fig. S6 Detection of mRNA transcripts in wing discs using the HCR assay. Confocal images displaying mRNA transcripts of *Lsd-2* (orange; A'-D'), *Lsd-1* (green; A"-D"), and *Acox57D* (red; A'''-D''') in the same discs as shown in Fig. 5A and Fig. 5B, respectively. (A-A'''/C-C''') Single focal plane; (B-B'''/D-D''') Z-stack projection; (a-d) BFP; merged images are shown in A-D. The dorsal/ventral (D/V) boundary and the anterior/posterior (A/P) boundaries are shown in C. Genotypes: (A/B) *en-Gal4/+; UAS-BFP/+*; and (C/D) *en-Gal4/UAS-Axn*^{RNAi}*; UAS-BFP/+*. Scale bar in panel (d): 20 μm.

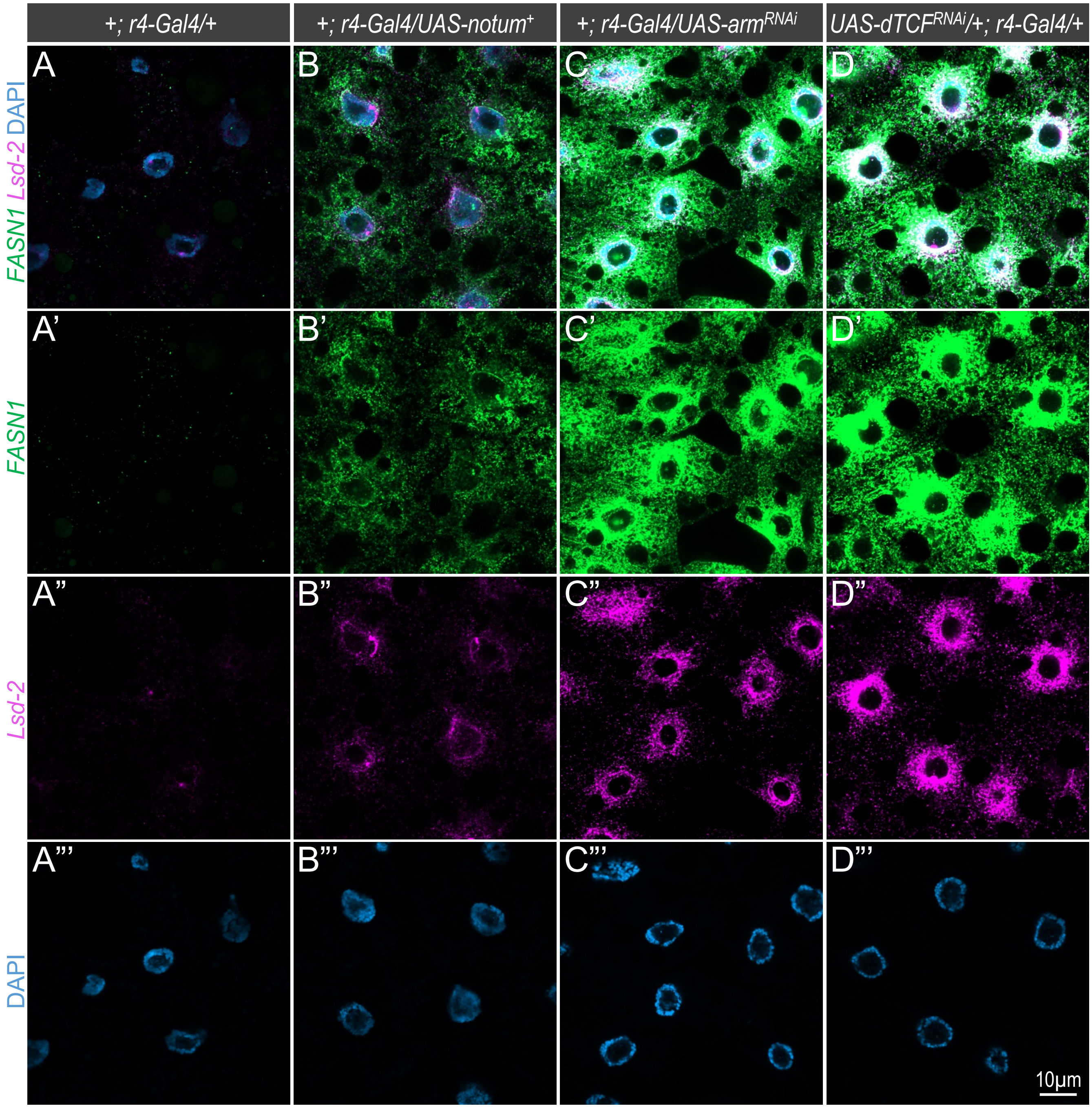

Fig. S7 Physiological role of Wnt signaling in regulating lipid metabolism-related gene expression in larval adipocytes. (A-D) Merged confocal images showing mRNA transcripts of *FASN1* (green) and *Lsd-2* (magenta) in larval adipocytes of indicated genotypes using the HCR assay. Single-channel images: (A'-D'): *FASN1*; (A"-D") *Lsd-2*; and (A'''-D''') DAPI. Genotypes: (A) +*; r4-Gal4*/+; (B) +*; r4-Gal4/UAS-notum*$^{+}$; (C) +*; r4-Gal4/UAS-arm*^{RNAi}; and (D) *UAS-dTCF*^{RNAi}/+*; r4-Gal4*/+. Scale bar in panel D''': 10 µm.

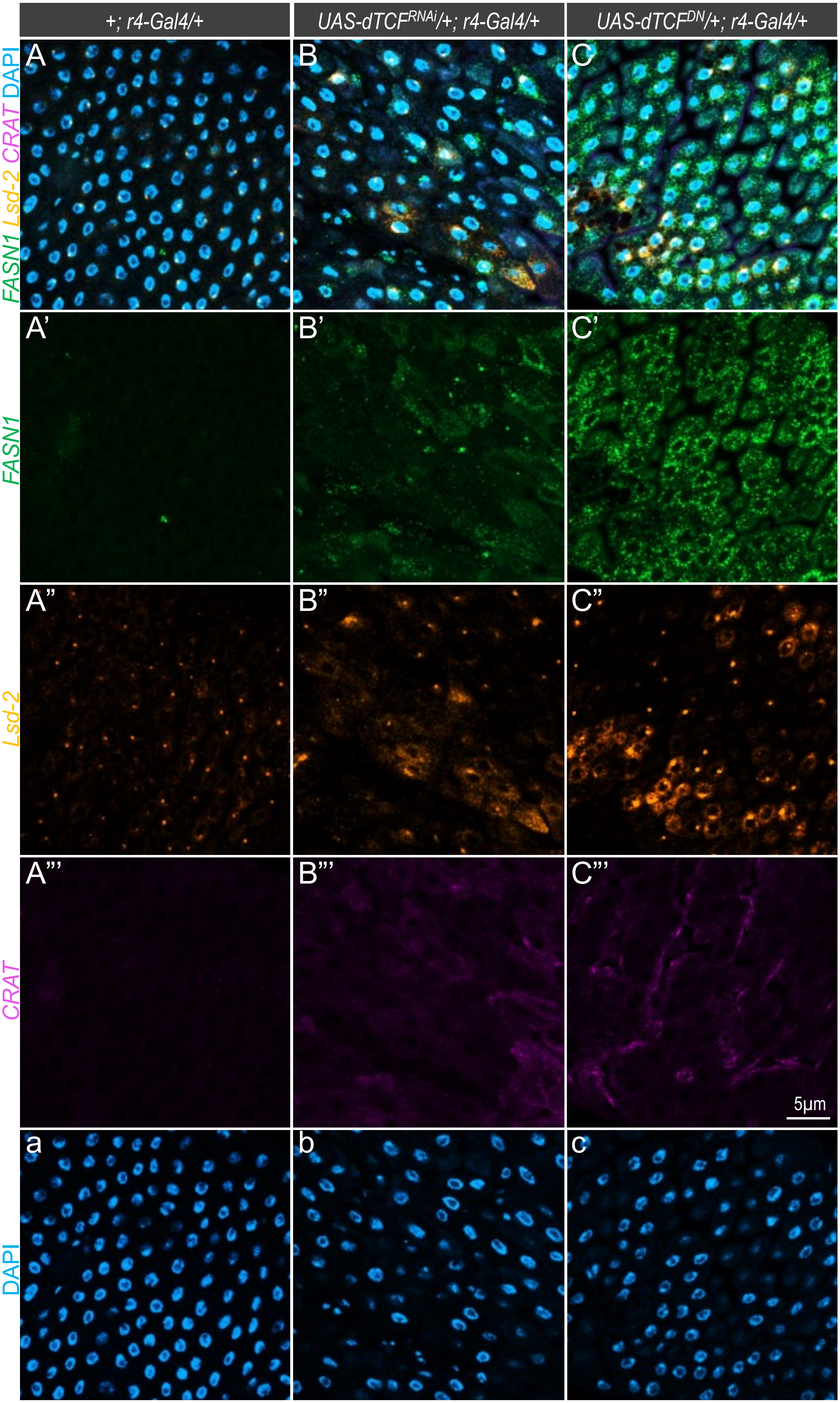
+; r4-Gal4/+
UAS-dTCF^RNAi/+; r4-Gal4/+
UAS-dTCF^DN/+; r4-Gal4/+
FASN1 Lsd-2 CRAT DAPI
FASN1
Lsd-2
CRAT
DAPI
A
B
C
A'
B'
C'
A''
B''
C''
A'''
B'''
C'''
a
b
c
5μm

Fig. S8 Detection of mRNA transcripts in adult midgut using the HCR assay. (A-C) Merged confocal images presenting mRNA transcripts of *FASN1* (green), *Lsd-2* (orange), and *CRAT* (magenta) using the HCR assay in the R2 region of adult midgut of the following genotypes: (A) *+; r4-Gal4/+*; (B) *UAS-dTCF*^{RNAi}*/+; r4-Gal4/+*; (C) *UAS-dTCF*^{DN}*/+; r4-Gal4/+*. Nuclei are stained with DAPI (blue). The scale bar in panel C’”: 5 μm.

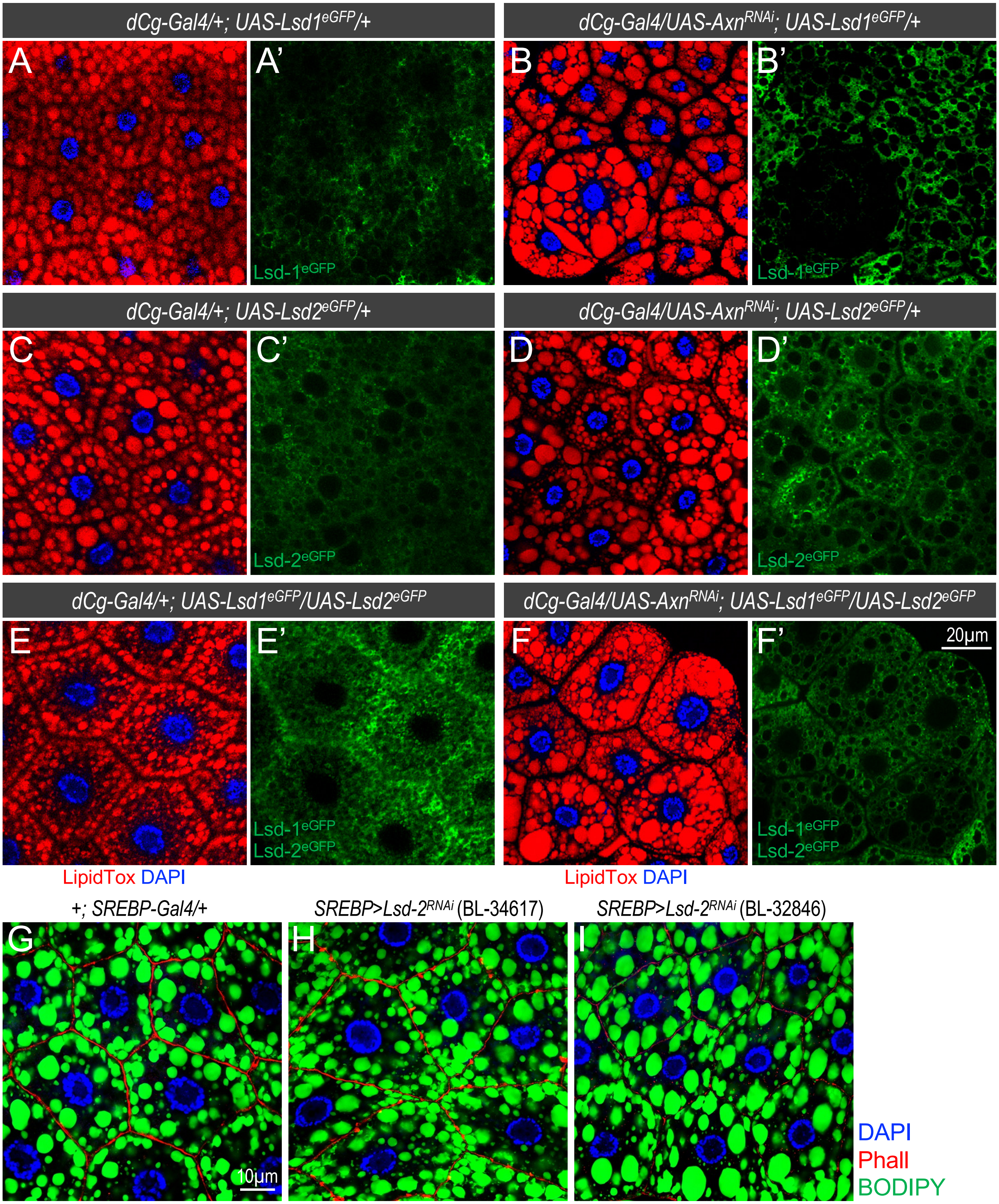

Fig. S9 Confocal images of larval adipocytes from the indicated genotypes. (A-D) Larval fat bodies stained with DAPI (blue) and LipidTox (red). Genotypes: (A/A') *dCg-Gal4/+; UAS- Lsd1^{eGFP}/+*; (B/B') *dCg-Gal4/UAS-Axn^{RNAi}; UAS-Lsd1^{eGFP}/+*; (C/C') *dCg-Gal4/+; UAS- Lsd2^{eGFP}/+*; (D/D') *dCg-Gal4/UAS-Axn^{RNAi}; UAS-Lsd2^{eGFP}/+*; (E/E') *dCg-Gal4/+; UAS- Lsd1^{eGFP}/UAS- Lsd2^{eGFP}*; and (F/F') *dCg-Gal4/UAS-Axn^{RNAi}; UAS- Lsd1^{eGFP}/UAS- Lsd2^{eGFP}*. The scale bar in panel F' applies to images A-F: 20 µm. (G-I) Larval fat bodies stained with DAPI (blue), BODIPY (green), and Phall (red). Genotypes: (G) *+; SREBP-Gal4/+*; (H) *+; SREBP-Gal4/UAS-Lsd2^{RNAi}* (BL-34617); and (I) *+; SREBP-Gal4/UAS-Lsd2^{RNAi}* (BL-32846). The scale bar in panel G applies to images G-I: 10 µm.

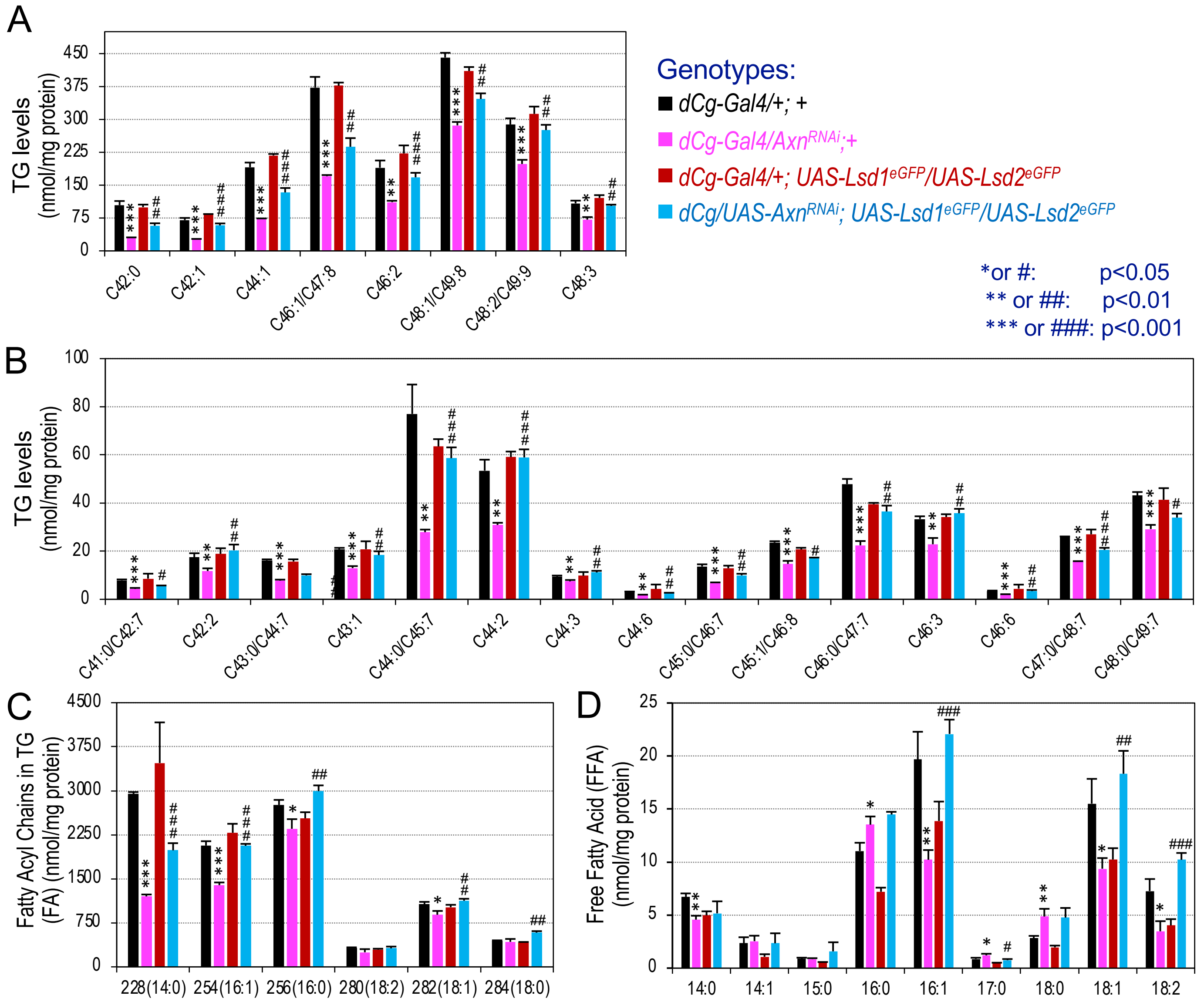

Fig. S10. Quantitative lipidomics measurement in fat body from third instar larvae. These figures present additional results from quantitative lipidomics analysis, focusing on various types of lipids in fat body dissected from third instar larvae. (A/B) Triglyceride (TG) levels, (C) Amount of fatty acyl chains within the total TG pool, and (D) FFAs contents. Specific genotypes are color coded and shown in panel A. Asterisks (*) represent comparisons between '*dCg-Gal4/+; +*' and '*dCg-Gal4/UAS-Axn^{RNAi}; +*', while pound signs (#) indicate comparisons between '*dCg-Gal4/+; UAS-Lsd1^{eGFP}/UAS-Lsd2^{eGFP}*' and '*dCg/UAS-Axn^{RNAi}; UAS-Lsd1^{eGFP}/UAS-Lsd2^{eGFP}*'. Statistical significance: * or #: p<0.05; ** or ##: p<0.01; *** or ###: p<0.001; these significance values are based on one-tailed unpaired *t*-tests.

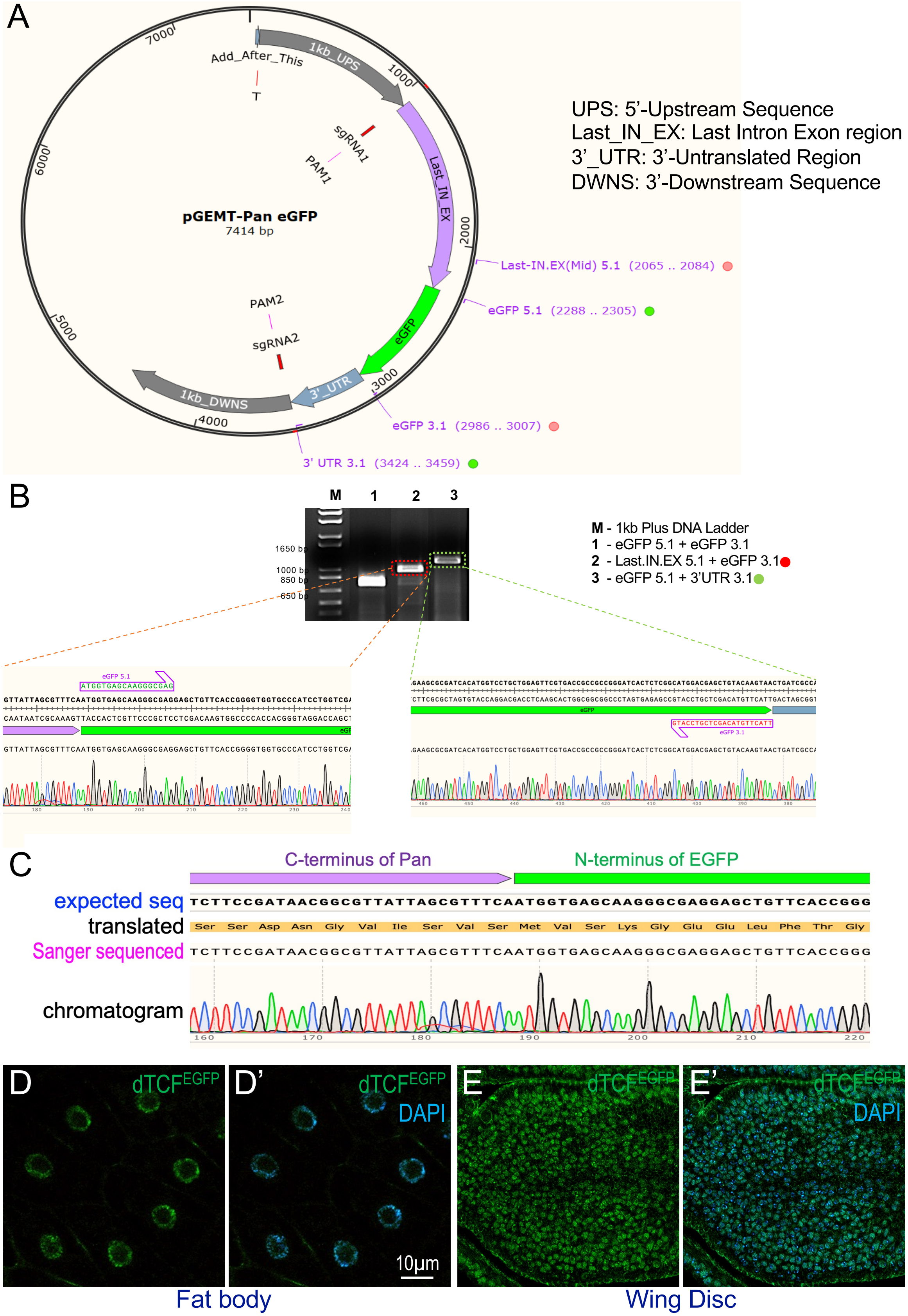

Fig. S11. Generation and validation of the *dTCF*^{EGFP} *Drosophila* strain. This figure outlines the process of creating and validating the *dTCF*^{EGFP} *Drosophila* strain. (A) The design of the donor template using the *pGEM-T-dTCF*^{EGFP} vector. (B) The validation of the *dTCF*^{EGFP} line by PCR using genomic DNA from *dTCF*^{EGFP} homozygous larvae. (C) Sequencing results confirming the presence and accuracy of the dTCF^{EGFP} in the genomic DNA. (D/D') Localization of dTCF in the nuclei of larval adipocytes. (E/E') Localization of dTCF in the nuclei of wing imaginal disc cells. (D'/E') Merged images with DAPI staining of nuclei (blue). The scale bar in panel D' applies to images D/D' and E/E': 10 μm.

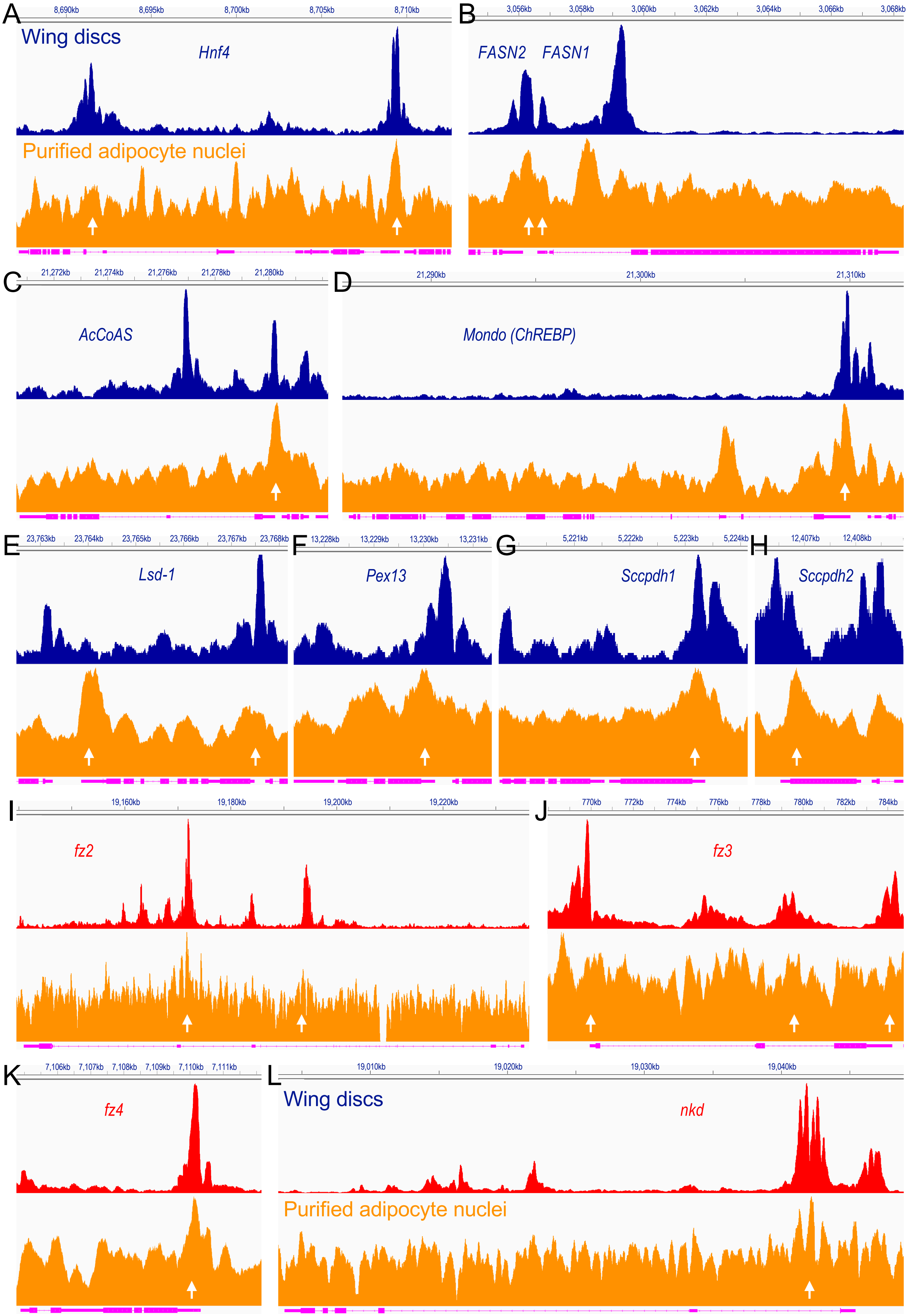

A
8,690kb
8,695kb
8,700kb
8,705kb
8,710kb
Wing discs
Hnf4
Purified adipocyte nuclei
B
3,056kb
3,058kb
3,060kb
3,062kb
3,064kb
3,066kb
3,068kb
FASN2
FASN1
C
21,272kb
21,274kb
21,276kb
21,278kb
21,280kb
AcCoAS
D
21,290kb
21,300kb
21,310kb
Mondo (ChREBP)
E
23,763kb
23,764kb
23,765kb
23,766kb
23,767kb
23,768kb
Lsd-1
F
13,228kb
13,229kb
13,230kb
13,231kb
Pex13
G
5,221kb
5,222kb
5,223kb
5,224kb
Sccpdh1
H
12,407kb
12,408kb
Sccpdh2
I
19,160kb
19,180kb
19,200kb
19,220kb
fz2
J
770kb
772kb
774kb
776kb
778kb
780kb
782kb
784kb
fz3
K
7,106kb
7,107kb
7,108kb
7,109kb
7,110kb
7,111kb
fz4
L
19,010kb
19,020kb
19,030kb
19,040kb
Wing discs
nkd
Purified adipocyte nuclei

Fig. S12

Fig. S12. Visualization of dTCF binding at genomic loci in CUT&RUN assay data from wing discs and purified adipocyte nuclei samples. To facilitate comparison, the tracks are color-coded as follows: blue tracks represent genes downregulated by Wnt signaling in the wing disc sample, red tracks indicate genes stimulated by Wnt signaling in the wing disc sample, and orange tracks depict data from the purified adipocyte nuclei sample. Arrows highlight overlapping peaks observed in both sample sources, with a noticeable increase in background noise peaks in the purified adipocyte nuclei sample. The y-axis is autoscaled, while multiple transcript isoforms are collapsed and presented in magenta beneath each respective track. Specific genes included are *Hnf4* (A), *FASN1* (B), *AcoCoAS* (C), *Mondo* (D), *Lsd-1* (E), *Pex13* (F), *Sccpdh1* (G), *Sccpdh2* (H), *fz2* (I), *fz3* (J), *fz4* (K), and *nkd* (L).

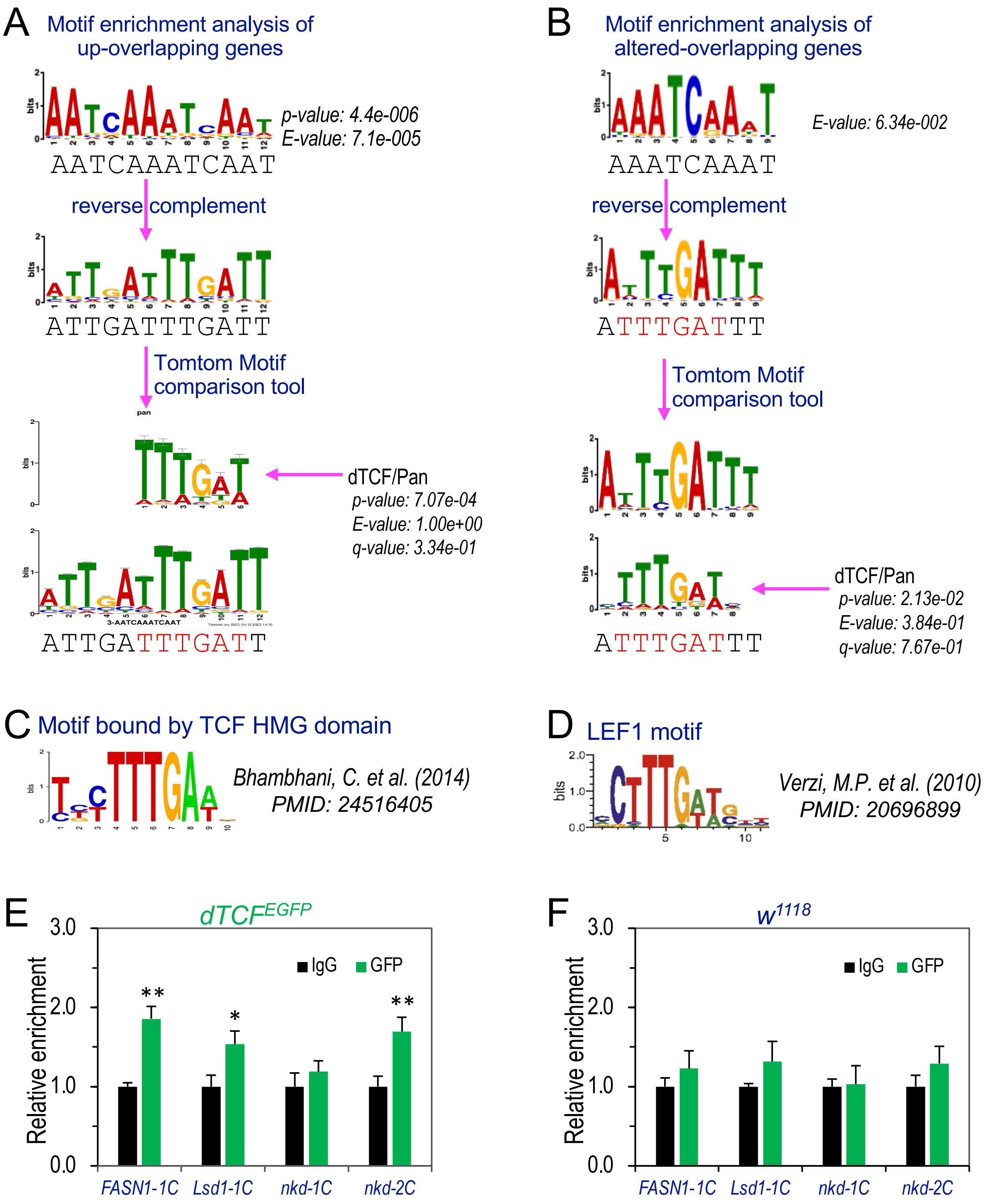

Fig. S13. Motif enrichment analyses of genes with called dTCF/Pan binding peaks. (A, B) Identification of a dTCF/Pan motif based on motif enrichment analysis of upregulated genes in both *Axn*^{RNAi} and *slmb*^{RNAi} adipocytes (A), and all called peaks in genes altered in both *Axn*^{RNAi} and *slmb*^{RNAi} adipocytes (B). For comparison, screenshots of motif bound by TCF HMG domain (C; from PMID 24516405) and LEF2 motif (D; from PMID 20696899) are included. (E, F) Validation of the CUT&RUN results using the ChIP-qPCR assay. (E) Relative enrichment of dTCF^{EGFP} was assessed on the promoters of Wnt target gene *nkd*, and lipid metabolism-related genes, *FASN1* and *Lsd-1*. Samples were from *dTCF*^{EGFP} homozygous embryos, with the IgG control shown in black. The primer pair '*nkd-1C*' was positioned away from the dTCF^{EGFP}-binding peaks ('*nkd-2C*') in the *nkd* promoter region. (F) Negative controls used *w*^{1118} embryos.

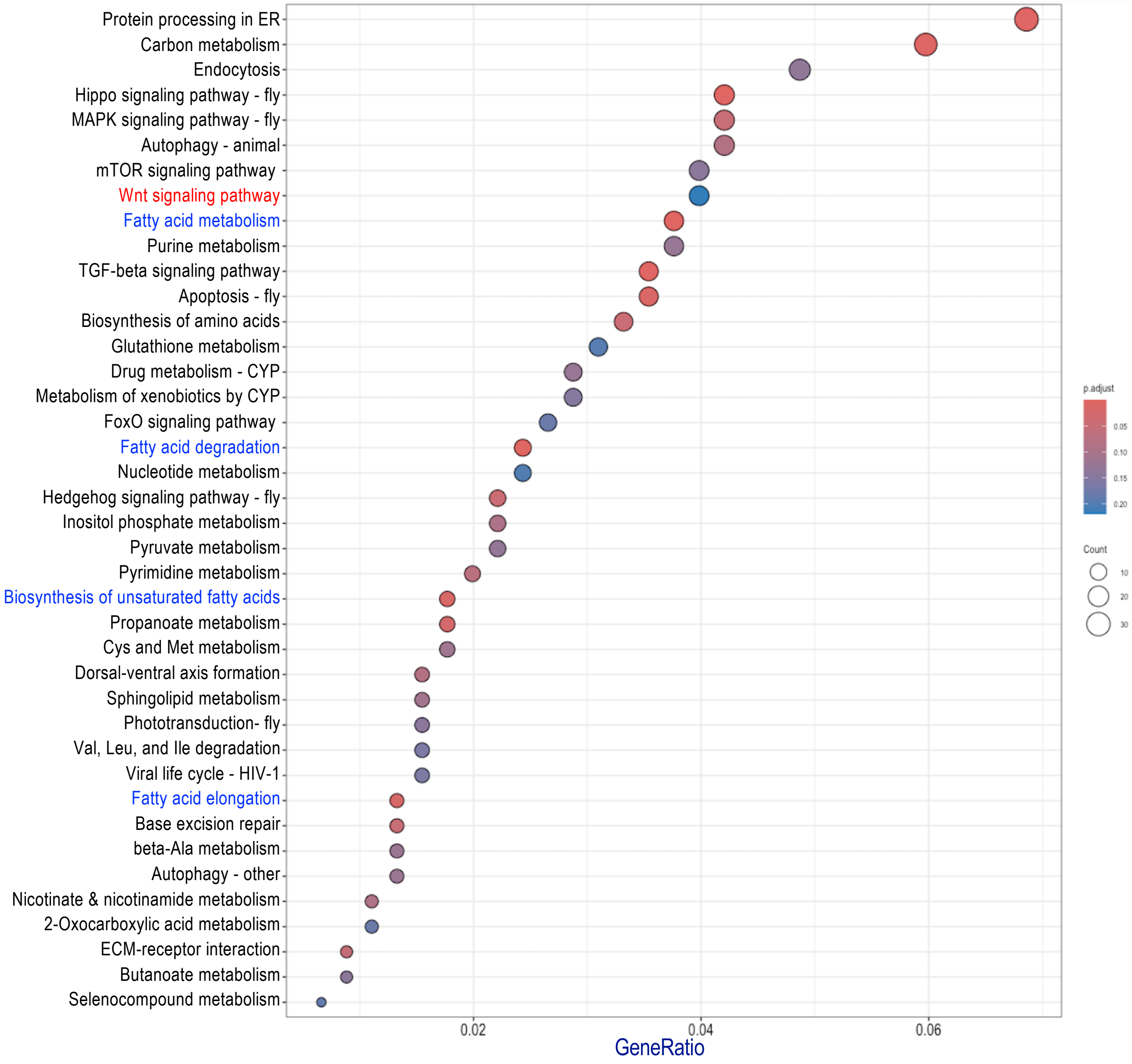

Fig. S14. Pathway enrichment analyses of genes altered in both *Axn*^{RNAi} and *slmb*^{RNAi} adipocytes with called dTCF/Pan binding peaks. This analysis identified the Wnt signaling pathway and several pathways related to lipid metabolism, which are highlighted.

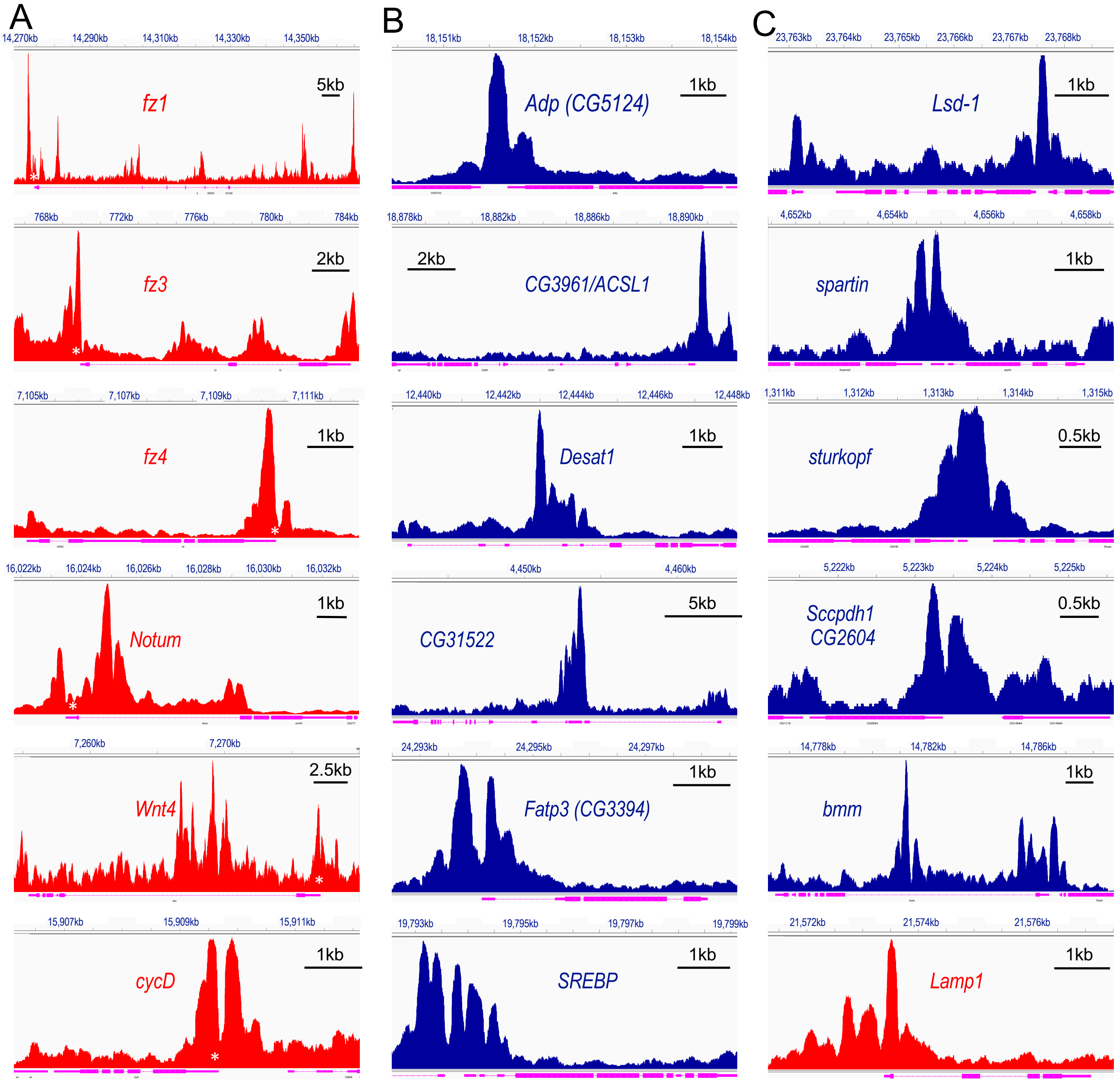

Fig. S15. dTCF/Pan binding at diverse genomic loci. This figure provides a visual representation of dTCF binding at various genomic loci, organized by their functional pathways. Genes activated by Wnt signaling are shown in red tracks, while genes downregulated by Wnt signaling are displayed in blue tracks. (A) Genes related to the Wnt signaling pathway. (B) Genes related to fatty acid biosynthesis pathway. (C) Genes related to LDAPs and lipid droplets. The asterisk (*) indicates the transcription start site (TSS). The y-axis is autoscaled, while different transcript isoforms are consolidated and displayed in magenta beneath each respective track.

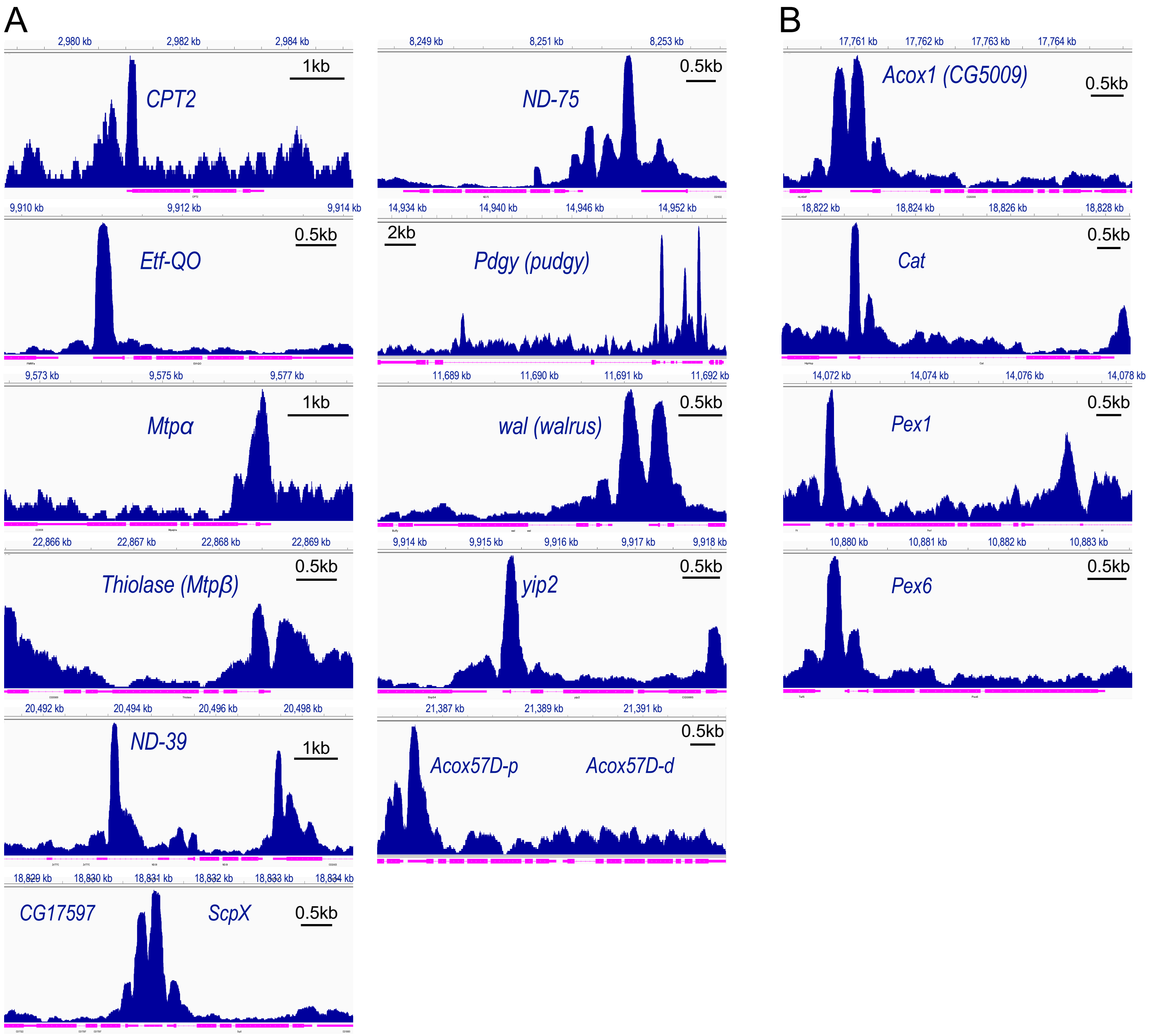

Fig. S16. dTCF/Pan binding at diverse genomic loci. (A) Genes related to FAO and the electron transport chain. (B) Genes related to peroxisome. Genes downregulated by Wnt signaling are displayed in blue tracks. The y-axis is autoscaled, while different transcript isoforms are consolidated and displayed in magenta beneath each respective track.

Table S1 List of the *Drosophila* stocks used in this study

Stock #	Genotype	Comments
7011	*w[1118]; P{w[+mC]=Cg-GAL4.A}2*	*dCg-Gal4*
31705	*y[1] v[1]; P{TRiP.HM04012}attP2*	*UAS-Axn[RNAi]*
32846	*y[[1] sc* v[1] sev[21]; P{TRiP.HMS00629}attP2*	*UAS-Lsd-2[RNAi]*
33832	*y[1] w*; P{w[+mC]=r4-GAL4}3*	*r4-Gal4 (III)*
33986	*y[1] sc* v[1] sev[21]; P{TRiP.HMS00946}attP2*	*UAS-slmb[RNAi]*
34617	*y[[1] sc* v[1] sev[21]; P{TRiP.HMS01292}attP2*	*UAS-Lsd-2[RNAi]*
35004	*y1 sc* v[1] sev[21]; P{TRiP.HMS01414}attP2*	*UAS-arm[RNAi]*
38394	*y[1] w[*]; wg[Sp-1]/CyO; P{w[+mC]=GAL4-dSREBPg.K}A45/TM6B, Tb[+]*	*SREBP-Gal4 (III),* Used with classical genetic recombinations
38395	*y[1] w[*]; P{w[+mC]=GAL4-dSREBPg.K}A39; SREBP[189]/TM6B, Tb[1]*	*SREBP-Gal4 (II),* Used with classical genetic recombinations
40848	*y[1] v[1]; P{TRiP.HMS02015}attP40/CyO*	*UAS-dTCF[RNAi]* , Homozygous animals were used
59759	*y[1] w*; Mi{PT-GFSTF.1}nkdMI00209-GFSTF.1/TM3, Sb1Ser1*	*nkd[EGFP]*
60561	*y[1] w* Mi{PT-GFSTF.1}armMI08675-GFSTF.1*	*arm[EGFP]*
62434	*y[1] v[1]; P{TRiP.HMJ23888}attP40/CyO*	*UAS-Axn[RNAi]* , Balancer changed to CyO,Tb
65020	*y[1] sc* v[1[sev[21]; P{TRiP.HMC05894}attP40*	*UAS-Lsd-1[RNAi]*
	Sp/Cyo; fz3-RFP/TM6B<br>*UAS-notum-V5/Cyo*<br>*UAS-dTCF[DN](II)*	a kind gift from Dr. Yashi Ahmed
	UAS-Lsd1[EGFP]/TM3,Sb<br>*UAS-Lsd2[EGFP]/TM3,Sb*	a kind gift from Dr. Mathias Beller

Table S2 Primers used to generate the dTCF[EGFP] strain using the CRISPR-Cas9 technique

Primer name	Primer Sequence (5' to 3')	
1kb_UPS_Pan 5.1	ggcggccgcgggaattcgatAAGACGTTTATCAAACATGTTC	
1kb_UPS_Pan 3.1	aggatctcagATATAAGAGTTACAGAACAGATC	
Last_IN_EX_Pan 5.1	actcttatatCTGAGATCCTTGCTGCATG	
Last_IN_EX_Pan 3.1	tgctcaccatTGAAACGCTAATAACGCC	
eGFP_Pan 5.1	tagcgtttcaATGGTGAGCAAGGGCGAG	
eGFP_Pan 3.1	tggcgatcagTTACTTGTACAGCTCGTCCATG	*Mutated PAM sites
3(pr)_UTR_Pan 5.1	gtacaagtaaCTGATCGCCATGGATTTG	
3(pr)_UTR_Pan 3.1	aattaatggaTTTTGGCAAGTTGTGTCTAATTTTAAAATAAAATAC	
1kb_DWNS_Pan 5.1	cttgccaaaaTCCATTAATTAATGCCTCTCTATCACATATG	
1kb_DWNS_Pan 3.1	gccgcgaattcactagtgatTACATGGAATAAAGGCTATC	
SgRNA1_Pan 5.1	gtcGATCTGTTCTGTAACTCTTA	
SgRNA1_Pan 3.1	aaacTAAGAGTTACAGAACAGATC	
SgRNA2_Pan 5.1	gtcGTCCATTAATTAATGCCTCTC	
SgRNA2_Pan 3.1	aaacGAGAGGCATTAATTAATGGA	
1kb_UPS_Pan(Seq) 5.1	AAGACGTTTATCAAACATGTTC	
1kb_UPS_Pan(Seq) 3.1	ATATAAGAGTTACAGAACAGATC	
Last_IN_EX_Pan(Seq) 5.1	CTGAGATCCTTGCTGCATG	
Last_IN_EX_Pan(Seq) 3.1	TGAAACGCTAATAACGCC	
eGFP_Pan(Seq) 5.1	ATGGTGAGCAAGGGCGAG	
eGFP_Pan(Seq) 3.1	TTACTTGTACAGCTCGTCCATG	
3(pr)_UTR_Pan(Seq) 5.1	CTGATCGCCATGGATTTG	
3(pr)_UTR_Pan(Seq) 3.1	TTTTGGCAAGTTGTGTCTAATTTTAAAATAAAATAC	
1kb_DWNS_Pan(Seq) 5.1	TCCATTAATTAATGCCTCTCTATCACATATG	
1kb_DWNS_Pan(Seq) 3.1	TACATGGAATAAAGGCTATC	
1kb_UPS(Mid)_Pan(Seq) 5.1	GTATGTGTCGCGGCAGAGCG	
Last_IN_EX(Mid)_Pan(Seq) 5.1	CTATCATGCTAATCACTCGC	

Table S3 Primers used for the ChIP-qPCR assay

Primer name	Primer Sequence (5' to 3')
Rp49-5.1C	TACAGGCCCAAGATCGTGAA
Rp49-3.1C	TCTCCTTGCGCTTCTTGGA
dFASN1-5.1C	GCTCTCTTTGGCACCCACCTCC
dFASN1-3.1C	CGTGTAGCCACTACAAATTGCC
Lsd1-5.1C	AACGGACTGCAGTTTGAACC
Lsd1-3.1C	ATCGCACCATATATGACCCG
nkd-5.1C	CTTTGAAGCCCCAGAGAGAGATTTC
nkd-3.1C	GCATCTGCCAGCAATTAGTGTAC
nkd-5.2C	GACTTTCAGACAAACGGACGCAC
nkd-3.2C	CGCAAAGCGCAGCTTGGCAG
